# Predicting Functional Alternative Splicing by Measuring RNA Selection Pressure from Multigenome Alignments

**DOI:** 10.1371/journal.pcbi.1000608

**Published:** 2009-12-18

**Authors:** Hongchao Lu, Lan Lin, Seiko Sato, Yi Xing, Christopher J. Lee

**Affiliations:** 1 Molecular Biology Institute, Center for Computational Biology, Institute for Genomics and Proteomics, Department of Chemistry and Biochemistry, University of California Los Angeles, Los Angeles, California, United States of America; 2 Department of Internal Medicine, University of Iowa, Iowa City, Iowa, United States of America; 3 Department of Biomedical Engineering, University of Iowa, Iowa City, Iowa, United States of America; University of Chicago, United States of America

## Abstract

High-throughput methods such as EST sequencing, microarrays and deep sequencing have identified large numbers of alternative splicing (AS) events, but studies have shown that only a subset of these may be functional. Here we report a sensitive bioinformatics approach that identifies exons with evidence of a strong RNA selection pressure ratio (RSPR) —i.e., evolutionary selection against mutations that change only the mRNA sequence while leaving the protein sequence unchanged—measured across an entire evolutionary family, which greatly amplifies its predictive power. Using the UCSC 28 vertebrate genome alignment, this approach correctly predicted half to three-quarters of AS exons that are known binding targets of the NOVA splicing regulatory factor, and predicted 345 strongly selected alternative splicing events in human, and 262 in mouse. These predictions were strongly validated by several experimental criteria of functional AS such as independent detection of the same AS event in other species, reading frame-preservation, and experimental evidence of tissue-specific regulation: 75% (15/20) of a sample of high-RSPR exons displayed tissue specific regulation in a panel of ten tissues, vs. only 20% (4/20) among a sample of low-RSPR exons. These data suggest that RSPR can identify exons with functionally important splicing regulation, and provides biologists with a dataset of over 600 such exons. We present several case studies, including both well-studied examples (*GRIN1*) and novel examples (*EXOC7*). These data also show that RSPR strongly outperforms other approaches such as standard sequence conservation (which fails to distinguish amino acid selection pressure from RNA selection pressure), or pairwise genome comparison (which lacks adequate statistical power for predicting individual exons).

## Author Summary

Alternative splicing is an important mechanism for regulating gene function in complex organisms, and has been shown to play a key role in human diseases such as cancer. Recently, high-throughput technologies have been used in an effort to detect alternative splicing events throughout the human genome. However, validating the results of these automated detection methods, and showing that the minor splice forms they detected play an important role in regulating biological functions, have traditionally required time-consuming experiments. In this study we show that such regulatory functions can very often be detected by a distinctive pattern of strong selection on RNA sequence motifs within the alternatively spliced region. We have measured this “RNA selection pressure ratio” (RSPR) across 28 animal species representing 400 million years of evolution, and show that this metric successfully predicts known patterns of alternative splicing, and also have validated its predictions experimentally. For example, whereas high-RSPR alternative splices were found experimentally to undergo tissue-specific regulation in 75% of cases, only 20% of low-RSPR cases were found to be tissue-specific. Using RSPR, we have predicted over 600 human and mouse alternative splicing events that appear to be under strong selection. These data should be valuable for biologists seeking to understand the functional effects and underlying mechanisms of splicing regulation.

## Introduction

Global analyses of alternative splicing (AS) have established its importance in protein diversity and gene regulation in higher eukaryotes [1],[2]. Alternative splicing can regulate biological function by altering the sequence of protein products and modulating transcript expression levels [3]. Alternative splicing can modify binding properties, intracellular localization, enzymatic activity, protein stability or post-translational modifications[4]. Alternative splicing is often regulated in a tissue-specific manner [5] and can undergo important changes in disease states such as cancer [6],[7]. All of these illustrate that it is necessary to study the functional effects of alternative splicing to understand the complexity of biological system and human disease.

One major challenge is the identification of functional alternative splicing events. In general, experimental methods that can directly demonstrate a biological function for an AS event are time-consuming, ad hoc, and impractical on a genome-wide scale. By contrast, high-throughput methods for surveying the transcriptome, such as EST sequencing [8], microarrays [9] or deep sequencing [10], mainly enable detection of whether a given splicing event is “present” or “absent” in a sample. Genome-wide analysis of such datasets has produced large databases of detected alternative splicing events, but with limited guidance for biologists about which ones are likely to be functional. More importantly, a number of studies have shown that a significant fraction of these detected events are probably not functional [2],[11],[12]. In this context, biologists need improved ways of distinguishing AS events that are likely to have important biological functions, before initiating costly experiments, such as high-throughput studies of regulation [13],[14].

There are multiple aspects of function that can be assessed using bioinformatics. Many studies have used the independent observation of the same alternative splicing event in multiple species as evidence that it is functional [15]–[18]. By contrast, introduction of a STOP codon more than 50 nt from the last exon-exon junction is predicted to cause nonsense-mediated decay; such a splice form will not produce a functional protein product (although the AS event itself might still play an important role in regulating function by down-regulating the transcript level) [19]–. Mapping of the AS exon to known protein domains or structures has also been suggested as an indicator of useful biological function [23],[24]. Many studies have indicated that an AS sequence segment consisting of an exact multiple of 3 nt length is more likely to be functional, since it can be alternatively spliced without affecting the protein reading frame [25],[26]. Evidence of tissue-specific regulation (from EST or microarray data) has also been taken as evidence of function [27]–[30]. While all of these criteria have been shown to be useful indicators of “function”, it should be emphasized that no one method can adequately capture this ill-defined concept, precisely because it has many different aspects.

Functions that are important for reproductive success are subject to selection pressure, which can be defined as a reduction in the frequency of observed mutations relative to that expected under a neutral model. For example, sequence conservation both within alternatively spliced exons and in flanking introns has been cited as evidence of important regulatory motifs. One useful extension of this principle is to separate total conservation into non-synonymous sites (i.e. where mutations will change the amino acid sequence) vs. synonymous sites (where mutations leave the amino acid sequence unchanged). Whereas alternative exons show poorer conservation than constitutive exons by total conservation metrics like phastCons [31], several studies have reported that measures of synonymous mutations (Ks, or ds) drop dramatically in certain types of alternative exons [26],[32],[33], particularly those that show tissue-specific regulation. Unlike standard conservation, such Ks effects cannot be attributed to protein function, and have thus been used as a measure of “RNA selection pressure” for features such as splicing factor binding sites, RNA secondary structure etc. [26], [34]–[40].

With the rapid growth in complete genome sequences for animals and plants [41], the strategy of seeking to detect RNA selection pressure gains increasing power as a way of predicting strongly selected AS regions [42],[43]. Past applications of Ks to this problem typically relied upon comparing a single pair of related genomes (e.g. human vs. mouse). Given the high level of identity seen in such exon comparisons (around 87% for human vs. mouse), the number of synonymous mutations expected in an alternative exon (just based on size, with no RNA selection pressure) is low, perhaps ten or fewer. Even if the observed number of synonymous mutations were three-fold lower, i.e. three or fewer, implying strong RNA selection pressure, the result would not be statistically significant, due to the small number of counts being compared. For this reason, such studies have typically not tried to predict *which* individual AS exons are strongly selected, but instead to compare entire groups of exons, e.g. all “minor-form exons” vs. all constitutive exons.

However, large multigenome alignments such as the UCSC 28 vertebrate genome alignment could improve predictive power, by measuring Ks simultaneously in many separate species. This has two benefits. First, the dataset of mutation counts for any given exon is greatly increased (naively, by a factor of up to 20-fold compared with a single pair of species), increasing the statistical power for detecting real selection pressure cases. Second, the ability to discriminate spurious cases is enhanced by utilizing a much more diverse set of genomes: various types of artifacts that might occur in one genome (e.g. a mutation “cold-spot” in mouse evolution) are unlikely to be conserved over 28 genomes spanning 300 million years of vertebrate evolution (such conservation would in fact indicate consistently strong selection).

In this paper we present a robust method for applying this approach, combining multigenome alignment data and RNA selection pressure calculations. This approach enables the detection of statistically significant RNA selection pressure for each individual exon, providing a direct prediction of whether that AS event has been strongly selected during vertebrate evolution. We have tested these prediction using a wide variety of data including known sets of regulatory targets, large-scale EST and microarray data, and RT-PCR analysis of tissue-specific splicing regulation.

## Results

### RNA selection pressure analysis of *GRIN1*

**Figure 1:**
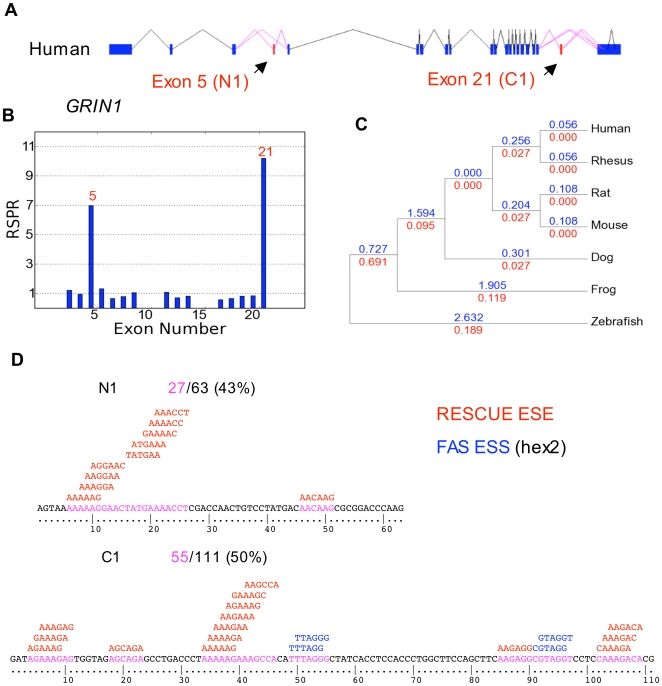
Prediction of functional alternative exons by RNA Selection Pressure Ratio. (A) The alternative splicing pattern of *GRIN1* in human; (B) RNA selection pressure ratio (RSPR) of each exon (measured vs. *all* conserved exons as the control set); (C) Synonymous mutation rates Ks for the constitutive exons (in blue) and the alternative exons (in red) on each branch of the phylogenetic tree. To fit the available space, only 7 of the 16 species in the computed tree are shown; the other species show the same pattern; (D) RESCUE ESE and FAS ESS predictions for the C1 and N1 exons.

As an initial test of the RNA selection pressure ratio (RSPR) metric, we applied it to *GRIN1*, a gene whose alternative splicing is well understood [44]. Of the 21 coding region exons, two exons (exons 5 and 21) show evidence of dramatically increased RSPR (RSPR values of 7 and 10 respectively) compared with the remaining exons (Figure 1). These two exons correspond to the well-studied N1 and C1 alternative exons, which have been shown to be regulated by PTB, NOVA2, hnRNP H and hnRNP A1 [45], and in turn control receptor desensitization [46] and NMDA receptor interactions [47]. The RSPR data indicate that these two exons have synonymous mutation rate approximately ten-fold lower than the surrounding exons (p-value  = 7.7×10^−25^). This very strong signal suggests that it should be possible to detect regulated alternative exons using the RSPR metric. These data suggest that more than half of the synonymous sites in the N1 and C1 exons are under some kind of negative selection pressure. This seems compatible with existing splicing factor motif databases. RESCUE-ESE [48] and FAS-ESS [49] predicted 43% of the N1 exon sequence and 50% of the C1 exon sequence as splicing factor binding sites (Figure 1D).

The *GRIN1* data provide evidence that using multigenome alignments (in this case *GRIN1* sequences from 16 species) can make RSPR more sensitive than simply comparing a pair of genomes. Figure 1C shows the synonymous substitution data (Ks) used to compute RSPR, annotated on the lineages leading to each species. The Ks values for the *GRIN1* N1 and C1 alternative exons are about ten-fold lower than the Ks values for the other exons, on each lineage. For example, within the primate lineage, these alternative exons showed a Ks level of 0.027 vs. 0.26 in the neighboring exons. Independent data for other mammal lineages (mouse, rat, dog) show a similar pattern, with total Ks values of 0.05 vs. 0.70 respectively. Moreover, frog, and zebrafish also show big decreases in Ks for the two alternative exons. These data indicate that the *GRIN1* N1 and C1 exons have been under a consistently strong negative RNA selection pressure for over 450 My. They also show why this computational approach can be more sensitive than simply comparing a pair of species, since each additional genome contributes further statistical evidence for the significance of this pattern.

### RSPR analysis of an alternative exon dataset of known NOVA targets

**Figure 2:**
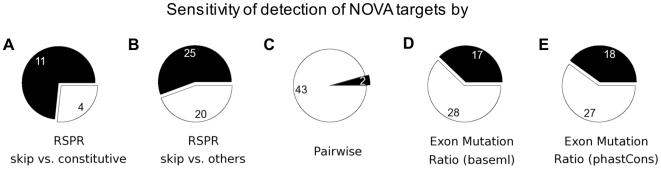
Sensitivity of detection of known NOVA splicing regulator targets by RSPR. Sensitivity of detection of known NOVA splicing regulator targets by five methods: (A) RSPR calculated for each alternative exon vs. constitutive exons as a control; (B) RSPR calculated for each alternative exon vs. all other exons in the gene; (C) RSPR calculated using just the human vs. mouse genomes; (D) mutation ratio calculated using baseml from the complete set of vertebrate genomes (see text); (E) sequence conservation calculated using phastCons from the complete set of vertebrate genomes (see text).

Next, to estimate the sensitivity of RSPR and compare it with existing methods, we applied it to a test set of alternative exons that are known targets of NOVA, generated by Jelen et al [50]. Using a conservative criterion (RSPR>3, P__RSPR_<0.001), RSPR detected 55% (25/45) to 73% (11/15) of these alternative exons as having strong RNA selection pressure (Figure 2; Table S1), depending on whether RSPR was computed for the target exon vs. all other exons in the gene, or vs. constitutive exons in the gene. An additional 24% (11/45) showed evidence of substantial selection pressure (RSPR>1.4) and had significant p-values, but fell below our cutoff of RSPR>3. To assess the specificity of RSPR, we also tested it on a dataset of alternative exons not known to be regulated. In the absence of a gold standard dataset of “true negative” exons that are known not to be targets of splicing regulation, we used a test set of 295 mouse alternative exons whose inclusion level remained relatively constant across a set of 10 different mouse tissues [51]. We detected RSPR>3 in only 9.5% (28/295) of these exons. This suggests that RSPR is relatively specific to exons that are targets of regulation.

We also tested two standard existing methods: pairwise genome comparison [52]; and standard measures of sequence conservation computed over the same multigenome alignments as the RSPR. RSPR differs from previous methods in combining two distinct approaches: first, instead of calculating simple sequence conservation, it separates evidence of nucleotide selection pressure from amino acid selection pressure (and ignores the latter); second, it measures this pressure across an entire family of aligned genomes. Ordinarily, studies of RNA selection pressure have compared a pair of genomes (e.g. human vs. mouse), but the pairwise method detected only 4% of the NOVA targets (Figure 2). Furthermore, even the successful pairwise predictions had much weaker p-values than from the multigenome RSPR calculation, by about 4 to 32 orders of magnitude. Secondly, we tested two standard measures of sequence conservation that are computed from all aligned genomes: baseml [53], and phastCons [54]. Baseml is calculated almost identically as RSPR, using the same software package (PAML), but unlike RSPR does not specifically estimate RNA selection pressure. With optimized signal-to-noise cutoffs, standard sequence conservation (baseml) detected 38% (17/45) of the NOVA targets, and phastCons detected 40% (18/45) of the NOVA targets.

### Validation of conserved alternative splicing in independent expression data from multiple species

**Figure 3:**
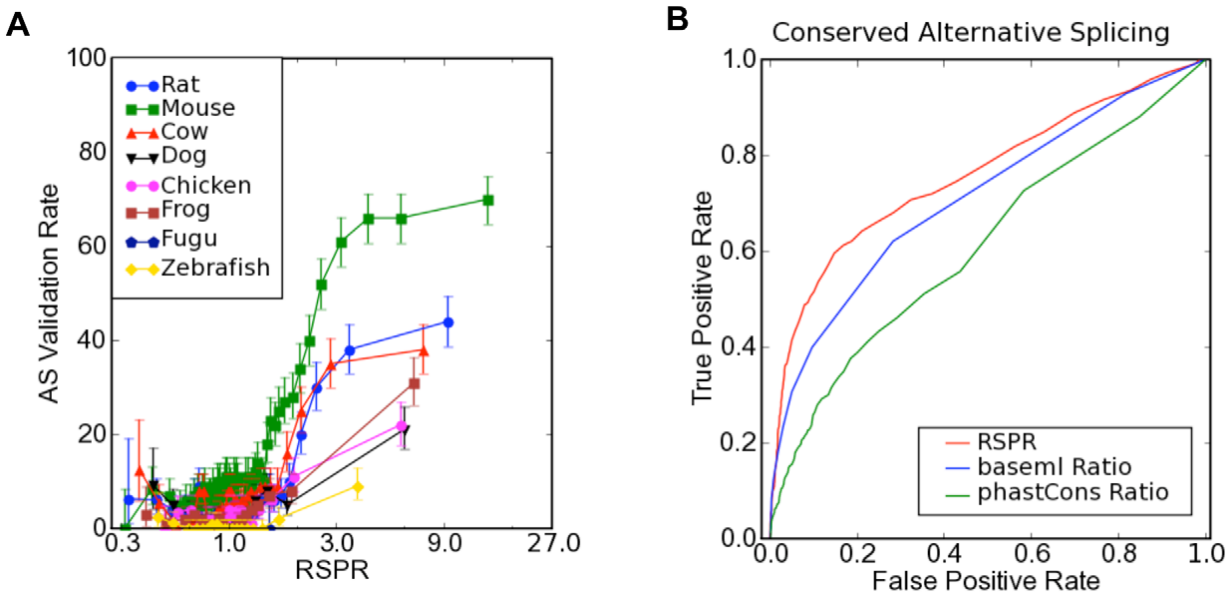
Validation of RSPR by independent alternative splicing data from other species. (A) The fraction of exons that were observed to be alternatively spliced in independent EST data for different species (y-axis), as a function of RSPR (x-axis). (B) The prediction performance (true positive rate vs. upper bound estimate of false positive rate) for RSPR vs. sequence conservation calculated using baseml or phastCons.

We next sought to compare RSPR's performance on a much larger dataset (Table 1). Numerous studies have reported that an alternative splicing event can be characterized as functional if it is independently observed in expression data from divergent species such as human and mouse [15],[16],[18]. We therefore tested each human exon in our dataset to see whether the orthologous mouse exon is observed to be alternative spliced in mouse EST data, and graphed the results as a function of the RSPR value (Figure 3A). For values of RSPR<1, only a small fraction of exons (approximately 10%) were also observed to be alternatively spliced in mouse, but the rate increased rapidly to 60–70% for values of RSPR of 3 or higher. These data suggest again that RSPR can help distinguish AS events that are likely to be functional, and that a large fraction of the exons with RSPR>3 are actually functional in both human and mouse. Furthermore, this pattern of validation can be seen not only in mouse EST data, but holds true in independent EST data from all of the vertebrate species in our study (Figure 3A). Although the total percentage of validation depends on the level of EST coverage for each organism (highest in mouse; lowest in zebrafish), the pattern is consistent in every EST dataset from mammals, chicken, frog, or zebrafish: high RSPR values predict which exons' alternative splicing will be conserved in other species. These results are consistent with the analysis by Plass and Eyras [39]. Throughout all species, an RSPR value of 3 appears to be a threshold for predicting strongly selected alternative splicing events.

**Table 1:** RSPR analysis of a dataset of 4626 human and 1935 mouse alternative exons.

		Whole				RSPR>3.0 & P__RSPR_<0.001			
		Major	Medium	Minor	Total	Major	Medium	Minor	Total
Human	Exons	3543	656	427	4626	85	108	152	345
	Genes^a^	2567	585	366	3063	84	103	139	301
Mouse	Exons	1361	346	228	1935	61	80	121	262
	Genes^a^	1151	312	191	1498	61	77	109	226

a Genes were classified by inclusion level according to the inclusion level of the alternative exon within that gene.

We have also used the AS validation rate in mouse to estimate the true positive vs. false positive rates for RSPR (Figure 3B). We classified exons that were independently observed to be alternatively spliced in mouse EST data as “true positives” (validated), and exons that were not observed to be alternatively spliced in the mouse EST data as “false positives” (not validated). We graphed the rate of true positives vs. false positives both as a function of increasing RSPR (Figure 3B). These data show that RSPR outperforms standard measures of conservation (e.g. 60% true positive rate for RSPR vs. 50% true positive rate for baseml vs. 40% true positive rate for phastCons, at the 20% false positive point) over the whole range of error rates. It should be emphasized that the actual false positive rate is likely to be significantly less, because mouse EST coverage is poor, resulting in many AS events that fail to be observed simply due to insufficient EST sampling.

**Figure 4:**
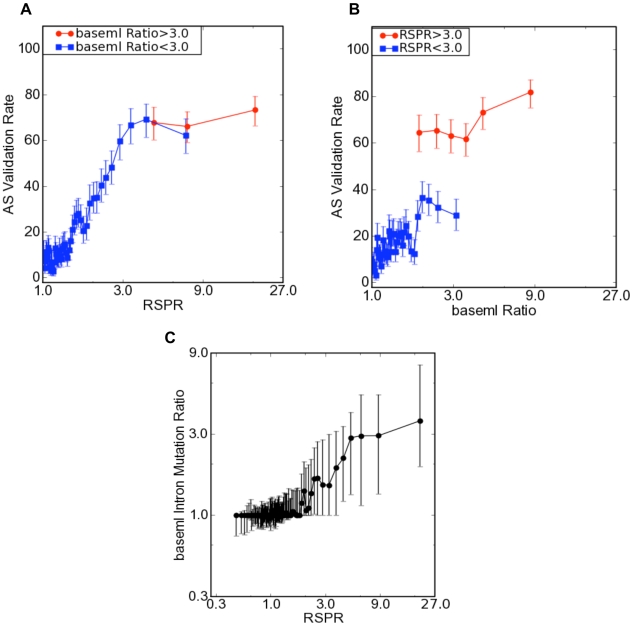
Comparing the predictive value of RSPR with standard sequence conservation. (A) The fraction of exons that were observed to be alternatively spliced in independent mouse EST data (y-axis), as a function of RSPR (x-axis), under different mutation ratio constraints (see text) (B) The fraction of exons that were observed to be alternatively spliced in independent mouse EST data (y-axis), as a function of baseml exon mutation ratio (x-axis), under different RSPR constraints (RSPR>3, high RNA selection pressure; RSPR<3, low RNA selection pressure). (C) The baseml intron mutation ratio (based on standard sequence conservation in the adjacent 50 nt of intron flanking the alternative exon; y-axis), as a function of RSPR (x-axis). Error bars represent one standard deviation.

To further assess the robustness of RSPR predictions, we subdivided the exons based on whether they showed standard conservation or not (measured using baseml), and evaluated the predictive power of RSPR in both cases (Figure 4A). RSPR showed a high level of predictive power both for exons with strong standard conservation (baseml mutation ratio>3) and weak conservation (mutation ratio<3). Thus RSPR is robust in predicting functional alternative splicing (regardless of the exact baseml value; Figure 4B ).

We have also found that it is possible to combine RSPR (measured from the exon sequence) with conservation measured in the flanking introns (Figure 4C). We computed the intron mutation ratio in the 50 nt on each side, using baseml. Exons with low RSPR showed flanking intron mutation ratios around one (i.e. neutral selection). By contrast, exons with high RSPR (RSPR>3) nearly all display higher levels of conservation in their flanking introns. This difference was statistically significant (p-value <10^−300^). On average, the amount of RNA selection pressure measured within the exon (RSPR) is matched by a proportional amount of selection pressure in the flanking 50 nt (intron mutation ratio). However, the intron mutation ratio displays a large variance (as shown by the error bars). These data suggest that exon-based RSPR can be complemented by the independent signal supplied by intron conservation, to improve prediction of strongly selected AS.

We also analyzed the effect of unusually small trees (only 5–9 species; Figure S1B) and of trees containing only placental mammal species (Figure S1C) compared with trees spanning both placental mammals and more distantly related vertebrates (Figure S1D). These data showed that the RSPR calculated using only 5–9 species successfully predicts whether the exon will be observed to be alternatively spliced in another species (in this case, mouse), just as we previously showed for our complete dataset (Figure S1B). Similarly, the RSPR calculations that only used placental mammal species showed the same direct correlation between RSPR and the AS validation rate. It should be noted that these are both unusual cases in our dataset: 86% of the RSPR calculations used 10 or more species, and 78% of the RSPR calculations spanned both placental mammals and more distant vertebrate species (Figure S1A). Finally, it should be emphasized that the highest RSPR values (those significantly above RSPR = 3) come almost exclusively from the cases with broad species representation, as one can see by comparing the RSPR range for the Placental Mammals set (Figure S1C) vs. the set that spans both Placental Mammals + Other Vertebrates (Figure S1D).

### Prediction of a set of Alternative exons with strong RNA selection pressure

We calculated RSPR for a set of 4626 alternative exons in human and 1935 exons in mouse, and applied a threshold of RSPR>3 and P__RSPR_<0.001, yielding 345 exons in human (vs. 4.5 expected by random chance) and 262 exons in mouse (Table 1 and Table S2). Some of the alternative splicing events detected by RSPR have already been demonstrated to be functional by experimental studies, such as *LRP8* [55], *SYK* [56], *DGKH* [57] and *WT1* [58] (Table S3). For example, the alternative exon in *LRP8* (ApoER2) encodes a 13 amino acid insertion containing a furin cleavage site important for *LRP8* function. *SYK* alternative splicing appears to play an important role in cancer; the exon-skip form (Syk(S), deleting 23 amino acid residues) occurs frequently in primary tumors but never in matched normal mammary tissues. *DGKH* has been shown to produce two splice forms, DGKH1 and DGKH2 which differ in the inclusion of a serile alpha motif (SAM) domain [57]. DGKH2 was detected only in testis, kidney and colon while DGKH1 was ubiquitously distributed in various tissues. *WT1* is a transcriptional regulator with an alternative exon encoding a 17 amino acids insertion, which appears to play a role in regulating cell survival and proliferation.

### Experimental validation using RT-PCR, EST and microarray data

**Figure 5:**
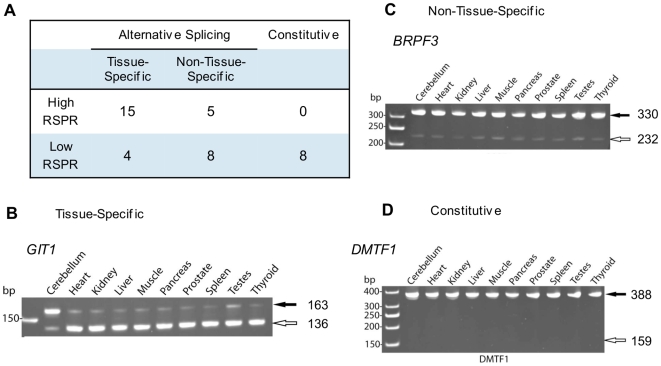
Experimental validation of RSPR predictions. Tissue-specific splice regulation is one indicator of functional alternative splicing that can easily be assayed experimentally. (A) Summary of RT-PCR results for 20 high-RSPR exons and 20 low-RSPR exons (see text). (B) RT-PCR detection of *GIT1* splicing in 10 human tissues shows that this alternative exon is regulated in a brain-specific manner. (C) Example of RT-PCR detection of non-tissue-specific AS. (D) Example of RT-PCR detection of constitutive splicing.

First, we performed RT-PCR experiments to test a subset of the RSPR predictions (Figure 5; Table S4). Tissue-specific splicing is one type of functional regulation that can be assayed easily in a panel of different tissue samples. We therefore extracted a random sample of 20 high-RSPR exons and 20 low-RSPR exons, and assayed their splicing by RT-PCR in cerebellum, heart, kidney and seven other human tissues. We classified an alternative exon as tissue specific if it was observed to be expressed as the major-form in at least one tissue, and as a minor-form in at least one other tissue. 75% (15/20) of the high-RSPR exons were found to be regulated in a tissue specific manner in this panel, vs. only 20% (4/20) among the low-RSPR exons (p-value 6.1×10^−4^). Thus a large fraction of high-RSPR exons were validated by experimental evidence of regulation. Low RSPR may indicate a lower probability of functional AS regulation; nearly half of the low-RSPR exons (8/20) did not even show evidence of alternative splicing in this panel (whereas the expected AS pattern was detected in this panel in 100% of high-RSPR exons (20/20).

We tested all the RSPR predictions using large-scale EST and microarray data. If RNA selection pressure is associated with exons whose splicing is tightly regulated (e.g. tissue-specific), then high-RSPR exons should not be expressed ubiquitously, but only in a subset of tissues or cells, and thus would be classified in EST datasets as minor-form (included in less than a third of a gene's transcripts) rather than major-form (included in more than 2/3 of transcripts). To test this hypothesis, we compared the ratio of major-form vs. minor-form exons in low-RSPR vs. high-RSPR groups (Table 1). Whereas the minor/major ratio was 0.0795 in the low-RSPR set, it rose to 1.79 in the high-RSPR set, more than a 20-fold increase. The ratio of intermediate-form over major-form also increased, from 0.158 in the low-RSPR set, to 1.27 in the high-RSPR set. Overall, only 24.6% of the high-RSPR exons were classified as major-form, vs. 74.7% in the low-RSPR set. A large fraction of exons in the high-RSPR dataset (44%) are annotated to be minor form. Thus, high levels of RSPR appear to be associated with exons that are included in a non-ubiquitous fashion, i.e. in a minority of a gene's transcripts.

To assess whether some of this pattern can be traced to tissue-specific splicing, we compared our RSPR results with a mouse microarray study that measured the inclusion level of mouse exons in 10 tissues [51]. Exons with a tissue-switched splicing pattern (i.e. minor-form in some tissue(s), but major-form in other tissue(s) [59] were three times as abundant in the high-RSPR set (28.2%, 11/39) than in the low-RSPR set (8.6%, 25/292). This difference was statistically significant, p-value = 0.0011 (Fisher exact test). We also saw a significant enrichment of tissue-specific exons detected by a previous EST analysis [5]. In the high-RSPR set, 9.3% (30/324) had strong evidence of tissue-specificity in the EST data (LOD>3), approximately double that observed in the low-RSPR set (5.2%, 204/3958). The p-value for this difference was 0.0026. To verify the EST analyses of tissue-specificity, we tested a sample of 10 high-RSPR exons with putative brain-specific splicing in the EST data, using RT-PCR in ten human tissues. 80% (8/10) showed tissue-specific splicing in this experiment (Table S5).

### RSPR analysis of minor-form, major-form, and intermediate-form AS exons

Numerous studies have examined the evolutionary patterns associated with different AS inclusion levels, such as minor-form, major-form, and intermediate form [60]–[63]. We have analyzed the RSPR distributions of these different forms of alternative splicing.

**Figure 6:**
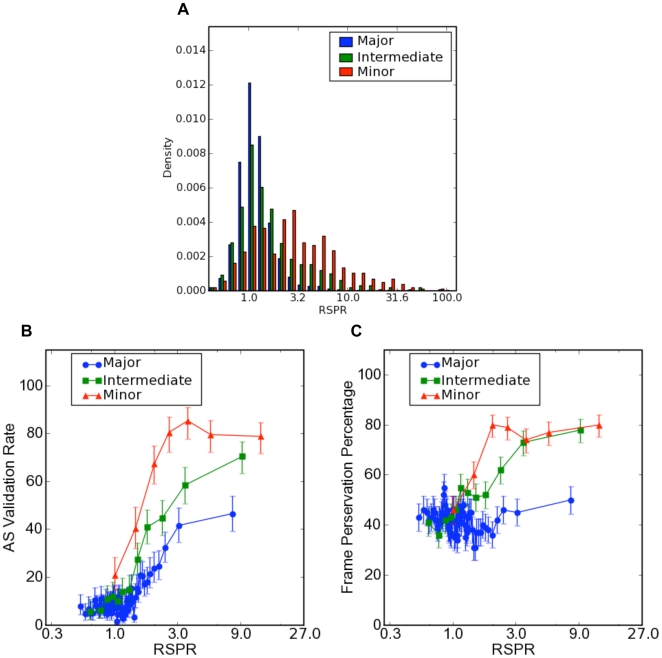
RSPR analysis of alternative splicing inclusion levels. (A) The distribution of the RNA selection pressure ratio (RSPR) for different inclusion levels (see text for detailed explanation); (B) The fraction of exons that were observed to be alternatively spliced in mouse EST data (y-axis) vs. RSPR; (C) The fraction of exons that preserve the protein reading frame, as a function of RSPR. Error bars represent one standard deviation.

Exons with different inclusion levels show strikingly different distributions of RNA selection pressure as measured by RSPR (Figure 6A). Major-form exons form a tight, symmetric distribution centered on RSPR = 1 (neutral selection). By contrast, minor-form exons display a broader distribution with a peak at RSPR = 3. At least within the constraints of this study (which was limited to exons that have been retained in mammalian genomes long enough to be found in multiple species, so that we can measure an RSPR value), 36% of conserved minor-form exons display strong RNA selection pressure, whereas only 2.3% of major-form exons had strong RSPR, which is in agreement with Xing et.al [26]. Intriguingly, intermediate-form exons revealed a bimodal distribution, with a main peak that closely follows the profile of the major-form distribution, and a small peak extending above RSPR = 3.

Validation by mouse EST data, and by frame-preservation, also highlight interesting differences between major- vs. minor-form exons. First, it is striking that high values of RSPR are predictive of functional AS (as measured by observation of alternative splicing of the orthologous mouse exon, in mouse EST data), at all three inclusion levels, even in major-form exons (Figure 6B). Thus, it appears that a small fraction of major-form exons are under RNA selection pressure for maintaining an important alternative splicing function. However, the total level of AS validation (by mouse EST data) was approximately two-fold higher for minor-form exons (over 80% for RSPR>3, vs. about 40% for major-form exons). Second, RSPR was predictive of functional AS at much lower values of RSPR for minor-form exons than for major-form exons. Third, frame-preservation revealed another interesting difference (Figure 6C). Whereas both minor- and intermediate-form exons displayed strong increases in frame-preservation with increasing RSPR, major-form exons remained at background frame-preservation levels (around 40%) across the whole range of RSPR values. This implies that RNA selection pressure is associated with “modular” protein sequence insertions for minor- and intermediate-form exons (i.e. insertions that do not alter the reading frame of the downstream protein sequence), but not for major-form exons.

### Prediction of a strongly selected AS event in the *EXOC7* gene

**Figure 7:**
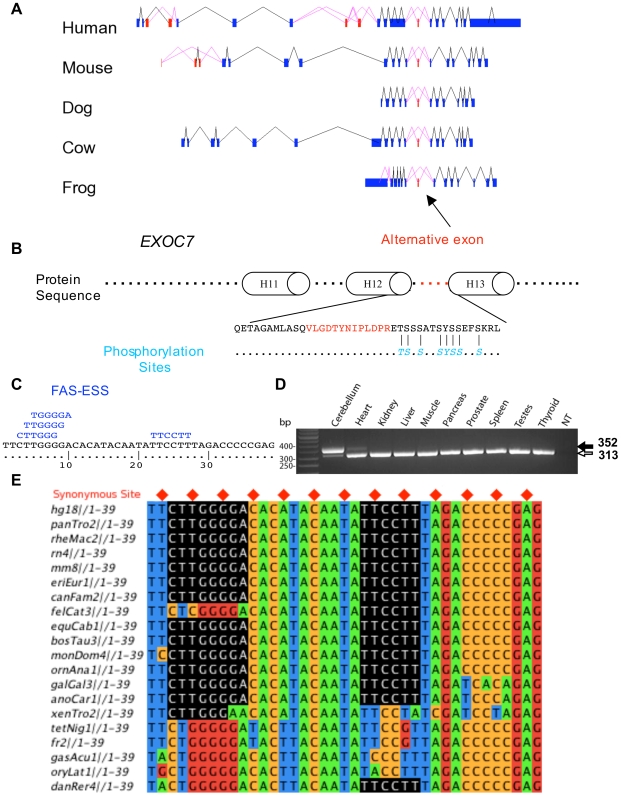
Prediction of a novel functional AS event in *EXOC7*. (A) RSPR detects strong selection in one AS exon (red), conserved in human, mouse, dog, cow and frog. (B) The exon encodes 13 aa in a loop region between helix 12 and helix 13, adjacent to a set of predicted phosphorylation sites. (C) Exonic Splicing Silencer (ESS) motifs predicted by FAS-ESS within the exon. (D) RT-PCR analysis of *EXOC7* splicing in 10 human tissues. (E) Conservation of ESS sequences (black) within the exon alignment.

Our RSPR results predict hundreds of alternative exons as strongly selected alternative splicing events. As one example, *EXOC7* is a component of the exocyst, an evolutionarily conserved octameric protein complex essential for exocytosis. The protein structure of mouse *Exoc7* [64], published recently, includes 19 α-helices linked with loops. EST data reveal several alternative exons, including two groups that map to disordered regions in the protein structure, one between helix 6 and helix 7, and the other between helix 12 and helix 13 (see Table S6). The latter (exon_id 25261 in ASAP II) contains 39 nucleotides, and is alternatively spliced within independent EST data not only for human, but also for mouse, dog, cow and frog (Figure 7). Our RSPR analysis detected this exon as having strong RNA selection pressure: RSPR = 6.24, measured over Human, Chimpanzee, Rhesus, Rat, Mouse, Hedgehog, Dog, Cat, Horse, Cow, Opossum, Platypus, Chicken, Lizard, Frog, Tetraodon, Fugu, Medaka and Zebrafish, with a P__RSPR_ of 1.67×10^−10^. ASAP2 reports this splicing event as tissue-specific to brain_nerve (with LOD 2.5) and retina (LOD 2.2) [5], and RT-PCR experiments confirmed its brain-specific splicing pattern (Figure 7D). NetPhos analysis [65] indicates a set of phosphorylation sites nearby (See Figure 7B). FAS-ESS (http://genes.mit.edu/fas-ess/) [49] identifies four ESS motifs in this exon (Figure 7E). Although the function of this exon is unknown, all these clues suggest that its alternative splicing has played an important functional role over a very long period of vertebrate evolution.

## Discussion

We have presented an effective method for estimating RNA selection pressure within an individual exon, and have tested its predictions against a variety of empirical measures of functional alternative splicing, such as known NOVA-regulated exons, conserved alternative splicing, frame preservation, and tissue-specific splicing patterns. We have also predicted a large dataset of strongly selected AS exons that can be useful targets for biologists to study the regulation of alternative splicing. Not only can the high-RSPR dataset furnish biologists with new insights into well-studied genes, but also identifies many new targets worthy of experimental study, in the form of strongly selected alternative splicing events.

These data suggest several possible benefits of RSPR. It provides a general way for distinguishing selection pressures that operate at the nucleotide level rather than protein level. RNA selection pressure may reflect many possible functional mechanisms, such as binding sites of splicing regulators including exon splicing enhancers (ESE) and exon splicing silencers (ESS) [48],[49]. As an example, ESE/ESS analyses for *GRIN1* and *EXOC7* annotated slightly under one-half of sites in these high-RSPR exons as ESEs or ESSs. Finally, RSPR integrates several powerful tools in comparative genomics, such as MULTIZ multiple genome alignments and PAML evolutionary model inference, and can in principle be applied to any genome.

Previous studies have reported that minor-form exons were associated with increased values of Ka/Ks and Ka compared with neighboring constitutive exons [26], [35]–[37] (for a review see [42]). Our results are consistent with this pattern; the distributions of Ka/Ks and Ka for minor-form exons showed significant increases relative to their control regions (neighboring consitutive exons; Figure S2 AB). Moreover, this pattern was also observed for minor-form exons with high RSPR values (Figure S2 C ).

Our approach has important limitations. First, it is important to emphasize that it seeks to detect RNA selection pressure, but gives no suggestion of what specific functional mechanism might cause it. Other possible patterns of selection that might be detected by RSPR include RNA secondary structure present within the pre-mRNA [42], miRNA binding sites, or binding sites in the parent DNA sequence. While some RSPR may be associated with ESE and ESS motifs, we cannot assume that they fully explain the RSPR in alternative exons. Consistent with several previous studies [35],[66] (see [43] for a review), we did not observe an overall correlation between the RESCUE-ESE density and RSPR, or between FAS-ESS density and RSPR in alternative exons (data not shown). Second, in this paper we have focused on demonstrating the predictive value of calculating RSPR within exonic sequence, without taking into account other useful information such as the flanking intron sequence, frame preservation, expression data from multiple species etc. An integrated prediction method would presumably make use of all available information [18]. A naive initial approach, based on simply multiplying the p-values from baseml (for the flanking intron conservation) and from codeml (for the exon RSPR conservation) did not appear to give major improvements of prediction accuracy in our preliminary tests, compared with simply using the codeml p-value. Since the two calculations use different programs (baseml vs. codeml) and different mutation models (single-nucleotide based vs. codon based), combining them in a single integrated calculation is not trivial. Third, RSPR is calculated based on the multiple genome alignment, and thus requires that an exon be sufficiently conserved among several genomes to be aligned.

## Materials and Methods

### Data

We obtained data for alternative exons (exon skipping) and constitutive exons in the same gene, from the ASAP II database [67]. Based on EST data, ASAPII classified each alternative exon as Major form (exon inclusion level greater than 2/3), Minor-form (exon inclusion level less than 1/3) and Intermediate-form (exon inclusion level between 1/3 and 2/3). We defined a constitutive exon as an exon that is included in all transcript isoforms of the gene (inclusion level 100%). We used several additional kinds of data to validate the prediction of functional alternative splicing, such as conserved alternative splicing based on independent EST data from multiple species assembled in the ASAPII database [67], tissue-switched alternative exons identified in the mouse microarray data of Pan et al. [51],[59].

We obtained all genome alignment information used in this study from the UCSC 28 vertebrate genome alignment hg18_multiz28way [68], available from ftp://hgdownload.cse.ucsc.edu/. This alignment includes the following complete genomes: Human, Armadillo, Bushbaby, Cat, Chicken, Chimpanzee, Cow, Dog, Elephant, Frog, Fugu, Guinea Pig, Hedgehog, Horse, Lizard, Medaka, Mouse, Opossum, Platypus, Rabbit, Rat, Rhesus, Shrew, Stickleback, Tenrec, Tetraodon, Tree shrew and Zebrafish.

We used the list of NOVA target exons of Jelen et al. [50] as a validation testset for our RSPR predictions. We were able to obtain genomic coordinates and genome alignments for 45 of NOVA targets published in Jelen et al., which were used for the validation tests presented in Figure 1D and Table S1.

### RNA selection pressure ratio calculation

We calculated the RNA Selection Pressure Ratio (RSPR) for each alternative exon compared with the constitutive exons within the same transcript isoform. Here we briefly summarize each step (See Figure S3): 1) each exon was mapped to orthologous exons in the 28 aligned genomes using the NLMSA alignment query tool [69] in the Pygr software package (http://code.google.com/p/pygr/). 2) each orthologous exon was required to retain the aligned splice sites and maintain a minimum of 70% amino acid identity (calculated by needle in EMBOSS [70]. For each exon, a minimum of 5 species was required. We ranked constitutive exons in a given gene by the number of species with orthologous exons, and identified the top third (i.e. most widely conserved exons), or a minimum of four constitutive exons, to represent that gene. 3) We then found the subset of species that were each aligned to all of these exons as well as to the alternative exon. That is, we computed the intersection of the sets of species that are aligned to each of these exons. This yielded the subset of species that we used for the RSPR calculation. 4) Next, we generated the list of constitutive exons that were aligned to all of these species, and used these as the control region for the RSPR calculation. 5) We extracted the alignment consisting of just this subset of species, for the control region + the alternative exon. 6) Prior to calculating RSPR, gaps were removed from the alignment. Specifically, only codons that were present in each of the species in the subset were retained in the alignment: amino acid sequences for the orthologous exons were aligned using clustalw [71], columns containing gaps were removed. This procedure ensures that RSPR is calculated using the exact same tree of species for the control region as for the alternative exon.

We used the multi-partition model D [72] of the PAML program codeml (http://abacus.gene.ucl.ac.uk/software/paml.html) to calculate maximum likelihood estimates of the RSPR, and a p-value for the null hypothesis of neutral RNA selection (i.e. RSPR = 1). Codeml uses a codon substitution model that is similar to the HKY85 nucleotide substitution model. Although codeml does not directly compute RSPR, its output parameters can be used to calculate RSPR. The gap-trimmed nucleotide sequences and a tree file including the phylogenetic tree for the subset of aligned species[68] were submitted to the codeml program, which estimates a set of evolutionary parameters from the whole tree, by maximum likelihood. We defined the RSPR as the ratio of synonymous mutation rate Ks for the alternative exon vs. the constitutive exons among all branches in the phylogenetic tree:
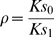
(1)where subscript 0 indicates the constitutive exons, and subscript 1 indicates the alternative exon. Based on this definition, a high RSPR value implies the alternative exon is under stronger negative RNA selection pressure than the corresponding constitutive exons. Ordinarily, for each branch in the tree, codeml estimates the total branch length *t* [73], which is related to the synonymous and non-synonymous substitution densities Ks and Ka via the ratio
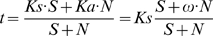
(2)where *S* and *N* are the number of synonymous and non-synonymous sites respectively. In multiple partition mode, *t_b_* for each branch *b* is replaced by *t_0b_* (for the constitutive exons) and *t_1b_* (for the alternative exon), related by the partition ratio
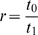
(3)which has a single value over the entire tree (i.e. for every branch *b*, *t_0b_* is constrained to be equal to *t_0b_* = *r t_1b_*). Based on the input phylogenetic tree and sequence alignment, codeml simultaneously estimates *t_1b_* (for each branch), *r* (for the whole tree), as well as the π_j_, κ_0_, κ_1_, and ω_0_, ω_1_ values (for the whole tree). Finally, combining equations 1, 2, and 3, we computed the RNA selection pressure ratio ρ:
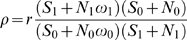
(4)where *S_0_, S_1_* and *N_0_, N_1_* are the number of synonymous and non-synonymous sites in the constitutive exons vs. the alternative exon, respectively.

We also modified codeml to be able to compute the likelihood under the constraint ρ = 1. We computed the p-value P__RSPR_ for the null hypothesis RSPR = 1 based on the log-odds ratio 2log(L(ρ_ML_)/L(ρ = 1)), which follows a χ^2^ distribution with one degree of freedom, where ρ_ML_ is the original maximum likelihood estimate of ρ obtained above.

### Sequence conservation scoring

We used two different standard methods for computing sequence conservation, baseml [53] and phastCons [54]. The baseml calculation used the HKY85 nucleotide substitution model (model = 4) and the Mgene = 3 multiple partition mode, similar to our codeml calculation. RSPR and baseml are calculated almost identically using the PAML package; the only difference is that whereas RSPR is calculated from the Ks ratio (eq. 1, above), for baseml we simply used the total nucleotide substitution ratio *r* (eq. 3, above). We calculated this ratio both for the alternative exon, and its flanking intronic regions (50 nt flanking each exon on each side).

PhastCons [54] is a widely used method for measuring sequence conservation in multiple genome alignments, used for example in the UCSC genome browser. We used phastCons to compute the ratio η for a region of interest compared with a control region (analogous to the RSPR), as follows:
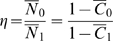
(5)where 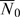 is the average probability of the phastCons non-conserved state in the control region, vs. 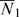 in the region of interest, and 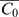 is the average phastCons score in the control region, vs. 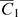 in the region of interest. We applied this both to exons (constitutive exons vs. alternative exon), and their flanking introns (50 nt flanking each exon on each side). We also calculated a P value based on the phastCons score for the null hypothesis that the mutation density is equal in the control region vs. region of interest. Specifically, we performed the Wilcoxon rank sum test on the phastCons scores for each nucleotide from the control region, vs. for each nucleotide from the region of interest.

For performing the NOVA analysis (Figure 2), we first determined cutoffs for the baseml ratio and phastCons ratio that yielded the same false positive rate in our ROC analysis (Figure 3B) as our RSPR cutoff (RSPR = 3): baseml ratio  = 2.9; phastCons ratio  = 45. Thus the NOVA analysis compares the sensitivity of these different methods, when calibrated to the same level of specificity.

### Validation sample construction

We generated a random sample of 20 high-RSPR exons (RSPR>3 and p<0.001), and a random sample of 20 low-RSPR exons (RSPR<1.0 and p<0.001). We then designed primers and performed RT-PCR as described below. As a separate test to confirm putative brain-specific splicing identified from EST data, we performed a join of the high-RSPR exon set and a previous database of EST evidence of brain-specific splicing [5]. We selected ten exons from this group, and performed RT-PCR as described below.

### RT-PCR and sequencing analysis of high RSPR exons in ten human tissues

Total RNA samples from 10 human tissues were purchased from Clontech (Mountain View, CA). Single-pass cDNA was synthesized using High-Capacity cDNA Reverse Transcription Kit (Applied Biosystems, Foster City, CA) according to manufacturer's instructions. For each tested exon, we designed a pair of forward and reverse PCR primers at flanking constitutive exons using PRIMER3. Two µg of total RNA were used for each 20 ul cDNA synthesis reaction. For each exon, 15 ng total RNA equivalent of cDNA were used for the amplification in a 10 µl PCR reaction. For each exon tested, three DNA polymerase systems were used to optimize RT-PCR reaction: Herculase® II Fusion DNA Polymerase (Stratagene, La Jolla, CA), HotStarTaq DNA Polymerase (Qiagen, Valencia CA) and Phire® Hot Start DNA Polymerase (NEB, Ipswich, MA). PCR reactions were run between 25 to 35 cycles (optimized for each exon) in a Bio-Rad thermocycler with an annealing temperature of 62 to 66°C (optimized for each exon). The reaction products were resolved on 2% TAE/agarose gels or 5% TBE polyacrylamide gels. Each result was a representation of 3–6 RT-PCR replications. DNA fragments with ambiguous sizes were cloned for sequencing using Zero Blunt® TOPO® PCR Cloning Kit (Invitrogen, Carlsbad, CA). Gel images (Table S4 and Table S5) were visually assessed as tissue specific if the alternative exon's splicing fraction (percentage of the exon-inclusion isoform vs. the exon-skip isoform) changed by a factor of two or greater in different tissues.

## Supporting Information

Table S1 RSPR for NOVA target exons with all other exons as control. RSPR results for known NOVA targets tabulated from Jelen et al. Significant RSPR results with RSPR >3.0 or P_RSPR<0.001 are marked in red.
(0.09 MB PDF)

Table S2 Complete Dataset of Alternative Exons with Strong RNA Selection Pressure in Human and Mouse. All human and mouse AS exons with RSPR>3 and P_RSPR<0.001 are listed.
(0.35 MB PDF)

Table S3 Confirmed functional alternative splicing in predicted dataset. RSPR predictions that were validated as functional AS events by published literature.
(0.12 MB PDF)

Table S4 RT-PCR Analysis of 20 High-RSPR AS Exons vs. 20 Low-RSPR AS Exons. No. 1–20 exons are with high RSPR (RSPR>3.0, P_RSPR<0.001), No. 21–40 exons are with low RSPR (RSPR<1.0, P_RSPR<0.001). Tissue-specific AS: T-AS, Non-Tissue-specific AS: NT-AS, Constitutive: Const; Solid arrow: Inclusion, Hollow arrow: Skipping, Dashed arrow: Non-specific amplification by PCR.
(3.20 MB PDF)

Table S5 RT-PCR Validation of 10 Brain-Specific Exons. We performed RT-PCR experiments to test a subset of the RSPR predictions. Tissue-specific splicing is one type of functional regulation that can be assayed easily in a panel of different tissue samples. We therefore extracted a subset of high RSPR exons that were also predicted by bioinformatics to up-regulated in brain based on available EST counts. We assayed their splicing by RT-PCR in cerebellum, heart, kidney and seven other human tissues. 80% (8/10) were found to be regulated in a tissue-specific manner. Thus a large fraction of high-RSPR exons were validated by experimental evidence of functional regulation.
(1.09 MB PDF)

Table S6 Alternative Exons in Exoc7. RSPR results for AS exons in Exoc7. TISSUE LOD: Log Odds score (log-base 10) for tissue-specific AS in the specified tissues.
(0.07 MB PDF)

Figure S1 Analysis of the species used for RSPR calculations on human AS exons. (A) A histogram of the number of species used for each RSPR calculation. (B–D) The fraction of exons that were observed to be alternatively spliced in independent mouse EST data (y-axis), as a function of RSPR (x-axis), for (B) the set of RSPR calculations with 5–9 species; (C) the set of RSPR calculation that used only placental mammal species; (D) the set of RSPR calculations that used both placental mammals and other vertebrates.
(0.61 MB TIF)

Figure S2 Ka/Ks and Ka distributions for different exon inclusion levels. For each alternative exon, we calculated the log-difference for its Ka/Ks or Ka relative to its control region (constitutive exons in the same gene). We then plotted their distributions for minor form, major form, and intermediate form alternative exons. (A) Histograms of the amino acid selection pressure (Ka/Ks); (B) Histograms of the non-synonymous mutation rate (Ka); (C) A scatter plot of the non-synonymous mutation rate (y-axis) vs. RSPR (x-axis).
(0.43 MB TIF)

Figure S3 A flow chart of the RNA selection pressure ratio (RSPR) calculation.
(0.07 MB TIF)
