# Supplementary material for: Predicting Functional Alternative Splicing by Measuring RNA Selection Pressure from Multigenome Alignments
Source: PLoS Comput Biol. 2009 Dec 18;5(12):e1000608. doi: 10.1371/journal.pcbi.1000608 (PMC2784930; doi:10.1371/journal.pcbi.1000608)
Supplement: Table S1 — RSPR for NOVA target exons with all other exons as control. RSPR results for known NOVA targets tabulated from Jelen et al. Significant RSPR results with RSPR >3.0 or P_RSPR<0.001 are marked in red. (0.09 MB PDF) [file pcbi.1000608.s001.pdf]

Table S1: RSPR for NOVA target exons with all other exons as control.

[a]The exons with RSPR >3.0 or P_RSPR<0.001 marked in red.

GENE NAME	EXON	EXON LENGTH	RSPR	P_{RSPR}
AGRN	31a	24	2.414	2.40×10^{-05}
ANK3	31	103	1.661	6.20×10^{-13}
APLP2	12a	36	3.72	2.50×10^{-07}
ATP13A4	7	135	1.154	2.60×10^{-04}
ATP2B1	34	154	13.285	2.60×10^{-39}
BCAS1	9	42	1.323	6.50×10^{-02}
BCAS1	10	168	1.023	1.30×10^{-05}
BCAS1	11	66	1.712	9.20×10^{-03}
BRD9	5	116	1.435	4.20×10^{-04}
CAMK2G	13a	33	5.838	1.40×10^{-07}
CAMK2G	13b	69	8.878	4.30×10^{-08}
CCDC9	1a	105	1.008	9.30×10^{-02}
CLASP1	9	117	3.248	1.10×10^{-05}
CLSTN1	10	57	3.458	1.60×10^{-07}
CP110	14	60	1.685	6.60×10^{-06}
CP110	14a	86	3.53	4.40×10^{-08}
DDR1	13a	111	7.353	1.50×10^{-23}
DNAJB5	1a	145	11.997	5.70×10^{-13}
EFNA5	3a	81	16.562	1.00×10^{-21}
EPB41L2	12a	168	1.671	3.10×10^{-04}
EPB41L2	12b	54	1.339	2.30×10^{-02}
EPB41L3	16	123	1.481	1.30×10^{-12}
EPHA5	4a	66	3.851	2.80×10^{-08}
GABRG2	9	24	9.227	6.40×10^{-09}
GLRA2	3a	68	2.954	1.70×10^{-13}
GPHN	7a	99	1.959	2.00×10^{-04}
GRIN1	19	111	8.912	1.80×10^{-23}
IGSF4A	8	84	5.432	2.20×10^{-08}
IGSF4B	2	102	6.124	9.00×10^{-13}
KCNQ2	13	30	0.755	1.30×10^{-01}
LRP1B	79	99	1.929	2.60×10^{-10}
MAP4K4	22a	192	6.136	2.10×10^{-12}
MAPK9	7	72	4.012	7.40×10^{-14}
MAPK9	6a	72	4.809	1.50×10^{-10}
NEO1	27	159	3.641	3.50×10^{-18}
NOVA1	4	72	6.701	5.40×10^{-08}
PLCB4	36a	37	3.335	6.80×10^{-08}
PTPRF	19a	33	56.183	4.20×10^{-08}
RAPLGA1	27	78	3.846	1.60×10^{-07}
SH2BPSM1	9	100	4.083	3.80×10^{-11}
SPNA2	4	60	4.599	2.80×10^{-14}
STXBP2	3	82	1.024	3.00×10^{-04}
TACC2	10	141	2.636	2.70×10^{-12}
TPM3	6	76	1.527	2.60×10^{-06}
TPM3	6a	76	1.661	7.70×10^{-03}
