# Supplementary material for: Predicting Functional Alternative Splicing by Measuring RNA Selection Pressure from Multigenome Alignments
Source: PLoS Comput Biol. 2009 Dec 18;5(12):e1000608. doi: 10.1371/journal.pcbi.1000608 (PMC2784930; doi:10.1371/journal.pcbi.1000608)
Supplement: Table S2 — Complete Dataset of Alternative Exons with Strong RNA Selection Pressure in Human and Mouse. All human and mouse AS exons with RSPR>3 and P_RSPR<0.001 are listed. (0.35 MB PDF) [file pcbi.1000608.s002.pdf]

Table S2. Complete Dataset of Alternative Exons with Strong RNA Selection Pressure in Human and Mouse.

A, Human (345 exons)

From total dataset, the exons with RSPR >3.0 and P_RSPR<0.001 are selected.

EXON_ID	LOCATION IN hg18	GENE SYMBOL	CLUSTER_ID	EXON LENGTH	RSPR	P_{RSPR}
87181	chr12:49732142-49732296	LETMD1	Hs.370457	155	3.284	2.00E-11
29234	chr22:16666577-16666627	MICAL3	Hs.528024	51	6.272	1.20E-10
87180	chr12:49732142-49732257	LETMD1	Hs.370457	116	4.695	1.10E-17
62195	chr17:39871565-39871609	KIAA0553	Hs.463129	45	10.612	5.00E-11
55380	chr3:185063235-185063283	PSARL	Hs.478469	49	4	2.40E-09
5795	chr11:62158358-62158423	GANAB	Hs.76847	66	5.988	3.10E-14
79783	chr16:24723527-24723673	TNRC6A	Hs.407740	147	7.086	6.80E-09
46901	chr10:123649371-123649499	ATE1	Hs.501239	129	3.534	6.60E-16
18868	chr13:41693400-41693530	DGKH	Hs.558670	131	3.71	4.00E-16
29950	chr14:104923067-104923099	PACS1L	Hs.525626	33	3.179	8.20E-05
54896	chr4:48091350-48091427	KIAA1458	Hs.479677	78	13.988	2.00E-09
98448	chr16:83673671-83673700	KIAA0513	Hs.301658	30	9.877	3.40E-04
51030	chr8:6569799-6569868	AGPAT5	Hs.490899	70	3.356	3.10E-11
62628	chr17:17701808-17701894	TOM1L2	Hs.462379	87	4.513	2.50E-19
67449	chr12:41031954-41032118	PPHLN1	Hs.444157	165	4.566	3.00E-09
74601	chr2:24361136-24361216	ITSN2	Hs.432562	81	7.994	1.00E-24
104545	chr1:43851026-43851058	PTPRF	Hs.272062	33	73.365	7.70E-13
25152	chr17:71598819-71598911	EXOC7	Hs.533985	93	5.347	3.90E-20
27222	chr13:31254813-31254884	LGR8	Hs.531002	72	12.532	4.80E-15
13263	chr2:135641733-135641753	RAB3GAP	Hs.570253	21	3.245	1.00E-07
25161	chr17:71594530-71594568	EXOC7	Hs.533985	39	6.243	1.70E-10
60811	chr19:14406402-14406466	PKN1	Hs.466044	65	4.623	2.80E-08
45650	chr11:74828033-74828047	PP1665	Hs.503297	15	20.259	1.90E-04
64971	chr22:49242329-49242406	SBF1	Hs.449098	78	3.669	2.30E-12
34387	chr6:30971160-30971270	DDR1	Hs.520004	111	7.191	5.70E-24
104536	chr1:43840329-43840355	PTPRF	Hs.272062	27	3.56	9.20E-07
81518	chr19:15370441-15370514	AKAP8L	Hs.399800	74	23.893	4.60E-12
99507	chr17:54998770-54998852	DHX40	Hs.29403	83	50.012	1.10E-08
68155	chr5:145828097-145828159	TCERG1	Hs.443465	63	30.823	1.10E-17
68162	chr5:145840906-145840956	TCERG1	Hs.443465	51	15.497	6.20E-07
53389	chr5:171273952-171274014	FBXW11	Hs.484138	63	3.234	1.60E-13
124382	chr22:19665036-19665056	FLJ30473	Hs.163543	21	4.137	6.90E-04
47031	chr10:105760564-105760656	SLK	Hs.500972	93	5.951	1.30E-12
58110	chr20:9405364-9405400	PLCB4	Hs.472101	37	3.216	3.30E-08
82148	chr6:161449973-161450063	MAP3K4	Hs.390428	91	22.123	1.90E-22
1286	chr4:89877646-89877729	FAM13A1	Hs.97270	84	3.327	4.10E-15
17236	chr6:168097987-168098022	MLLT4	Hs.567305	36	14.383	2.90E-13
39968	chr17:2217315-2217449	RUTBC1	Hs.513861	135	4.187	5.50E-25
68226	chr17:4736473-4736583	MINK1	Hs.443417	111	6.338	6.80E-26
47271	chr10:94228382-94228526	IDE	Hs.500546	145	5.186	7.80E-21
38681	chr19:6697040-6697207	TRIP10	Hs.515094	168	3.644	2.90E-14
118919	chr7:98486474-98486551	SMURF1	Hs.189329	78	4.847	1.40E-05
100103	chr5:154153553-154153753	LARP	Hs.292078	201	4.429	3.00E-31
100102	chr5:154153553-154153675	LARP	Hs.292078	123	3.133	1.30E-19

21808	chr17:77458372-77458425	PCYT2	Hs.551512	54	4.211	3.20E-06
117942	chr10:80740687-80740947	RAI17	Hs.193118	261	4.278	8.00E-33
74714	chr13:102101710-102101748	TPP2	Hs.432424	39	4.617	3.60E-18
52567	chr6:91310992-91311072	MAP3K7	Hs.485968	81	4.285	3.40E-11
61787	chr17:71481301-71481461	ACOX1	Hs.464137	161	3.889	2.80E-14
44388	chr12:103005894-103005946	HCFC2	Hs.506558	53	3.373	3.20E-06
124338	chr11:85378941-85379090	PICALM	Hs.163893	150	3.445	2.10E-05
32261	chr1:6089042-6089156	CHD5	Hs.522898	115	3.507	4.10E-18
87411	chr14:30879407-30879433	C14orf125	Hs.370299	27	9.383	2.40E-14
31910	chr1:24652485-24652581	DJ462O23.2	Hs.523442	97	17.647	1.90E-04
122250	chr19:758869-758902	PTBP1	Hs.172550	34	13.261	5.20E-06
19148	chr13:78825289-78825360	C13orf10	Hs.558528	72	8.927	2.60E-17
116778	chr16:113506-113544	C16orf35	Hs.19699	39	6.797	1.50E-12
31313	chr1:31973371-31973469	BAI2	Hs.524138	99	6.837	4.50E-11
118460	chr8:120653597-120653671	ENPP2	Hs.190977	75	6.98	4.80E-11
49176	chrX:18170480-18170631	SCML2	Hs.495774	152	3.266	1.60E-11
90410	chr1:209552685-209552926	RCOR3	Hs.356399	242	4.165	2.10E-13
26847	chr19:7876693-7876740	MAP2K7	Hs.531754	48	24.9	2.10E-14
79366	chr11:32394612-32394662	WT1	Hs.408453	51	3.733	1.30E-04
89748	chr1:157426185-157426286	IGSF4B	Hs.365689	102	6.124	9.00E-13
72142	chr4:113403593-113403691	C4orf16	Hs.435991	99	4.864	1.50E-10
44315	chr12:107462333-107462438	SART3	Hs.506663	106	4.178	8.10E-09
60630	chr19:43398833-43398883	DPF1	Hs.466651	51	4.25	9.40E-05
50959	chr8:17841831-17841947	PCM1	Hs.491148	117	5.051	2.00E-18
180	chr19:40898681-40898749	TZFP	Hs.99430	69	3.077	1.60E-04
50980	chr8:17885022-17885078	PCM1	Hs.491148	57	4.546	4.00E-08
8031	chr11:47703865-47703964	FNBP4	Hs.6834	100	3.12	4.00E-07
73112	chr8:38222908-38222975	DDHD2	Hs.434966	68	3.751	1.70E-06
66097	chr10:24865709-24865828	KIAA1217	Hs.445885	120	3.035	7.60E-12
99836	chr17:59065801-59065893	MAP3K3	Hs.29282	93	4.224	1.40E-14
66087	chr10:24823435-24823539	KIAA1217	Hs.445885	105	4.015	8.10E-16
34763	chr5:137530106-137530315	BRD8	Hs.519337	210	4.171	4.20E-21
68333	chr7:92664622-92664646	LOC253012	Hs.443169	25	4.675	2.00E-06
34768	chr5:137527675-137527721	BRD8	Hs.519337	47	3.97	4.60E-07
34761	chr5:137532058-137532276	BRD8	Hs.519337	219	8.878	1.60E-19
105424	chr2:54415458-54415491	FLJ40298	Hs.269546	34	3.716	2.10E-05
34764	chr5:137530106-137530198	BRD8	Hs.519337	93	5.109	8.90E-17
90163	chr14:103036246-103036290	MARK3	Hs.35828	45	3.9	2.10E-04
105274	chr9:114132542-114132575	ROD1	Hs.269988	34	13.413	1.70E-07
110199	chr9:34980665-34980809	DNAJB5	Hs.237506	145	4.566	7.40E-10
117912	chr2:127542209-127542301	BIN1	Hs.193163	93	4.136	6.80E-13
19639	chr5:61704022-61704135	KIF2	Hs.558351	114	4.78	1.40E-19
41198	chr15:42402455-42402509	H63	Hs.512867	55	3.034	4.20E-07
73631	chrX:149517714-149517808	MTM1	Hs.434285	95	4.738	3.50E-24
107971	chr14:74482669-74482731	PGF	Hs.252820	63	6.593	3.50E-09
50351	chr8:130960817-130960899	FAM49B	Hs.492869	83	3.248	9.30E-13
81247	chrX:46919362-46919435	RBM10	Hs.401509	74	27.528	9.20E-16
739	chr14:54273492-54273755	SAMD4	Hs.98259	264	6.1	3.80E-18
107193	chr7:97203547-97203600	TAC1	Hs.2563	54	5.079	5.90E-06
74272	chr16:2746336-2746608	SRRM2	Hs.433343	273	4.959	8.00E-21
50348	chr8:130961804-130961886	FAM49B	Hs.492869	83	3.151	3.50E-08

85686	chr6:109780683-109780810	C6orf185	Hs.373914	128	3.066	2.10E-07
83018	chrX:138523041-138523088	MCF2	Hs.387262	48	8.216	1.70E-08
4630	chr4:71826208-71826261	RIPX	Hs.7972	54	7.622	3.50E-07
46072	chr11:64297478-64297553	SF1	Hs.502829	76	23.362	5.30E-29
121430	chr4:37731122-37731241	TBC1D1	Hs.176503	120	6.422	4.20E-28
99789	chr2:55382512-55382712	KIAA1212	Hs.292925	201	5.616	1.40E-16
99788	chr2:55383709-55383792	KIAA1212	Hs.292925	84	7.506	1.90E-23
45726	chr11:72019770-72019831	PDE2A	Hs.503163	62	6.116	8.90E-06
19712	chr9:139179459-139179569	GRIN1	Hs.558334	111	8.921	7.70E-25
75150	chr1:1739136-1739174	GNB1	Hs.430425	39	13.484	5.10E-06
19695	chr9:139162425-139162487	GRIN1	Hs.558334	63	8.468	1.40E-13
49470	chr9:127144319-127144399	DKFZP434C212	Hs.495134	81	5.125	1.40E-16
17070	chr12:7246104-7246214	PEX5	Hs.567327	111	3.623	6.10E-13
98001	chr4:76798191-76798289	G3BP2	Hs.303676	99	6.681	3.70E-18
121196	chr10:76535425-76535632	DUSP13	Hs.178170	208	6.282	3.90E-18
95545	chr8:38276119-38276265	WHSC1L1	Hs.32099	147	7.291	2.40E-18
52641	chr6:86407729-86407888	SYNCRIP	Hs.485877	160	3.29	7.40E-08
1858	chr16:87181451-87181522	LOC124245	Hs.93670	72	4.519	3.60E-12
28282	chr2:233847106-233847162	APG16L	Hs.529322	57	15.583	5.90E-16
18162	chr2:165291862-165291900	COBLL1	Hs.560777	39	10.116	2.00E-07
104895	chr12:22556848-22556880	KIAA0528	Hs.271014	33	12.438	9.90E-08
104897	chr12:22546940-22547005	KIAA0528	Hs.271014	66	15.863	2.80E-16
27725	chr7:138709687-138709757	LUC7L2	Hs.530118	71	30.562	9.20E-16
59086	chr2:148385576-148385599	ACVR2	Hs.470174	24	4.988	2.40E-07
73768	chr5:139832008-139832064	ANKHD1	Hs.434219	57	12.244	3.30E-13
103124	chr2:39069915-39069959	SOS1	Hs.278733	45	4.604	1.00E-05
105334	chr4:56427795-56427815	SEC3L1	Hs.269665	21	6.608	1.80E-06
85477	chr12:37998142-37998270	KIF21A	Hs.374201	129	4.341	6.00E-16
112802	chr1:67167161-67167234	MI-ER1	Hs.21757	74	74.681	5.40E-15
92299	chrX:149633609-149633659	MTMR1	Hs.347187	51	6.532	1.00E-17
60325	chr19:54997603-54997668	AP2A1	Hs.467125	66	5.151	1.00E-08
79981	chr19:39397869-39398045	C19orf13	Hs.407368	177	4.141	2.50E-14
54026	chr5:64987219-64987236	FLJ13611	Hs.482301	18	47.941	3.30E-09
85475	chr12:38002737-38002951	KIF21A	Hs.374201	215	3.319	3.40E-04
62325	chr17:36052233-36052337	SMARCE1	Hs.463010	105	7.265	5.00E-19
112262	chr1:78163461-78163502	NEXN	Hs.22370	42	4.315	8.60E-07
85494	chr12:38026535-38026573	KIF21A	Hs.374201	39	17.877	2.80E-13
97635	chr1:90245492-90245897	ZNF326	Hs.306221	406	31.811	6.30E-43
97636	chr1:90245759-90245897	ZNF326	Hs.306221	139	16.883	1.90E-29
51073	chr7:158238233-158238295	CHR2SYT	Hs.490795	63	3.199	2.00E-12
52660	chr6:86305275-86305301	SNX14	Hs.485871	27	4.644	9.20E-07
94127	chr11:13423147-13423276	GMRP-1	Hs.332382	130	54.593	1.00E-25
73279	chr16:29920236-29920352	FLJ90652	Hs.434864	117	6.267	1.70E-14
29779	chr16:4670034-4670099	MGRN1	Hs.526494	66	9.838	8.00E-12
86611	chr7:94106589-94106696	SGCE	Hs.371199	108	7.688	1.10E-11
90507	chr1:36413086-36413196	FLJ10350	Hs.356096	111	3.059	2.60E-08
68242	chr17:27805883-27806008	PSMD11	Hs.443379	126	12.54	2.70E-08
40648	chr16:3303050-3303176	ZNF75A	Hs.513292	127	4.177	1.60E-12
108484	chr3:27395744-27395851	SLC4A7	Hs.250072	108	3.795	1.80E-12
116058	chr8:27155111-27155191	STMN4	Hs.201058	81	5.193	7.40E-09
53579	chr5:147786969-147787703	FBXO38	Hs.483772	735	7.755	1.10E-19

53578	chr5:147786969-147787478	FBXO38	Hs.483772	510	6.843	9.50E-20
39413	chr17:59154390-59154418	LYK5	Hs.514402	29	25.372	1.20E-11
16549	chr1:15728170-15728354	KIAA0962	Hs.567430	185	5.395	2.90E-08
67362	chr20:47900754-47900788	SLC9A8	Hs.444202	35	4.893	2.30E-08
26907	chr17:55394683-55394759	LOC51136	Hs.531701	77	15.567	6.90E-26
124602	chr15:66263022-66263059	PIAS1	Hs.162458	38	7.453	2.90E-07
47453	chr10:74474745-74474815	P4HA1	Hs.500047	71	3.623	5.60E-22
84422	chr1:60102085-60102128	HOOK1	Hs.378836	44	7.276	7.80E-09
85650	chr17:4826108-4826235	CAMTA2	Hs.373952	128	6.094	7.10E-13
61069	chr2:225365935-225366033	DOCK10	Hs.46578	99	3.823	5.00E-11
77527	chr19:54341235-54341261	PPFIA3	Hs.413748	27	5.786	1.60E-05
126046	chr6:122790747-122790800	HSF2	Hs.158195	54	5.601	1.20E-10
80934	chr18:21869793-21869885	SS18	Hs.404263	93	28.754	3.70E-11
85570	chr4:185566306-185566423	IRF2	Hs.374097	118	4.814	2.80E-17
104979	chr1:181782861-181783010	C1orf16	Hs.270775	150	6.407	3.30E-20
67839	chr6:119337292-119337438	C6orf60	Hs.443789	147	5.764	1.20E-30
49234	chrX:13735690-13735809	GPM6B	Hs.495710	120	5.442	4.30E-08
59660	chr2:61154097-61154190	KIAA1841	Hs.468653	94	5.675	2.70E-11
123802	chr11:57318058-57318129	CTNND1	Hs.166011	72	3.81	1.80E-17
36857	chr1:155191308-155191337	ARHGEF11	Hs.516954	30	4.819	4.50E-06
52366	chr6:132096421-132096556	ENPP3	Hs.486489	136	3.392	1.60E-11
114008	chr13:74796374-74796538	TBC1D4	Hs.210891	165	4.07	4.60E-09
102450	chr1:53487640-53487816	LRP8	Hs.280387	177	12.136	1.30E-20
102438	chr1:53510864-53510902	LRP8	Hs.280387	39	6.082	3.30E-04
52075	chr8:95774081-95774231	FLJ20171	Hs.487471	151	5.049	7.10E-24
9111	chrX:134938711-134938800	SLC9A6	Hs.62185	90	4.865	1.60E-07
53387	chr5:171317206-171317307	FBXW11	Hs.484138	102	25.768	6.10E-43
81389	chr20:52025335-52025376	BCAS1	Hs.400556	42	3.912	1.80E-06
34948	chr4:107052794-107052844	LOC255743	Hs.518921	51	10.861	2.20E-13
81392	chr20:52007378-52007443	BCAS1	Hs.400556	66	6.248	2.20E-07
29424	chr1:149549311-149549355	PIK4CB	Hs.527624	45	4.586	1.10E-10
32078	chr10:99502604-99502624	ZFYVE27	Hs.523194	21	3.06	4.10E-06
61133	chr19:5562131-5562641	SAFB2	Hs.465690	511	6.935	7.50E-17
55969	chr3:101513367-101513411	FLJ11046	Hs.477003	45	3.585	9.40E-10
75678	chr7:35679336-35679413	FLJ22313	Hs.424711	78	4.642	9.20E-16
34318	chr6:31964725-31964826	BAT8	Hs.520038	102	6.497	3.30E-21
60680	chr19:40444668-40444724	LISCH7	Hs.466507	57	3.904	5.30E-12
94668	chr5:150091641-150091661	DCTN4	Hs.328865	21	8.629	3.10E-10
62858	chr16:84225021-84225239	KIAA0182	Hs.461647	219	3.025	1.50E-23
52392	chr6:130412594-130412668	L3MBTL3	Hs.486466	75	17.275	3.10E-11
81517	chr19:15370441-15370577	AKAP8L	Hs.399800	137	23.028	2.50E-17
104476	chr4:38740574-38741050	KLHL5	Hs.272251	477	3.574	2.80E-15
40715	chr14:52588312-52588395	DDHD1	Hs.513260	84	6.045	9.20E-10
125020	chr10:13679651-13679677	PRPF18	Hs.161181	27	9.752	3.40E-06
55455	chr3:182171557-182171637	FXR1	Hs.478407	81	3.257	3.20E-10
20312	chr1:11641177-11641272	FBXO44	Hs.556006	96	23.947	5.20E-06
25693	chr10:103893997-103894054	PPRC1	Hs.533551	58	6.72	7.30E-05
73933	chr17:38978452-38978562	ETV4	Hs.434059	111	4.824	8.30E-12
17796	chr19:14138828-14138842	LPHN1	Hs.565302	15	49.931	5.50E-05
62457	chr17:27560482-27560577	RHOT1	Hs.462742	96	10.61	1.30E-11
62458	chr17:27562248-27562370	RHOT1	Hs.462742	123	4.441	8.30E-24

17797	chr19:14136433-14136517	LPHN1	Hs.565302	85	22.858	4.80E-16
84574	chr7:31101806-31101889	ADCYAP1R1	Hs.377783	84	8.828	4.00E-07
84576	chr7:31106266-31106349	ADCYAP1R1	Hs.377783	84	5.878	1.50E-15
51900	chr7:44662442-44662486	OGDH	Hs.488181	45	3.969	8.80E-13
48377	chr1:202773181-202773248	MDM4	Hs.497492	68	7.996	4.20E-10
106685	chr1:42444020-42444121	FOXJ3	Hs.26023	102	3.632	4.90E-16
100138	chr19:16348933-16349065	EPS15L1	Hs.292072	133	4.03	1.10E-21
42586	chr15:45846526-45846582	SEMA6D	Hs.511265	57	14.768	3.20E-16
36538	chr21:33866727-33866806	SON	Hs.517262	80	29.553	8.70E-19
34963	chr10:69388876-69388899	HERC4	Hs.51891	24	11.88	1.50E-05
123710	chr3:38148091-38148133	ACAA1	Hs.166160	43	4.486	9.90E-04
99293	chr1:9739126-9739155	CLSTN1	Hs.29665	30	13.558	4.30E-13
42587	chr15:45848070-45848237	SEMA6D	Hs.511265	168	5.778	2.10E-25
75630	chrX:129116812-129116942	PDCD8	Hs.424932	131	4.087	1.10E-12
53647	chr5:137382543-137382734	C5orf5	Hs.483473	192	5.209	1.00E-13
53658	chr5:137320065-137320130	C5orf5	Hs.483473	66	7.446	2.70E-11
4408	chr4:144598283-144598372	GAB1	Hs.80720	90	4.088	3.20E-19
102530	chr16:19467338-19467423	CP110	Hs.279912	86	8.745	1.20E-13
99977	chr4:110622282-110622386	SEC24B	Hs.292472	105	7.369	9.40E-20
103385	chr17_random:470851-470945	M17S2	Hs.277721	95	3.514	5.20E-04
124509	chr22:41602838-41602960	PACSIN2	Hs.162877	123	3.839	6.30E-19
79520	chr1:234772838-234772963	LGALS8	Hs.4082	126	7.238	7.00E-11
65939	chr4:48199602-48199619	KIAA0826	Hs.446102	18	13.247	9.60E-11
93274	chr4:41335706-41335741	KIAA1102	Hs.335163	36	3.407	1.50E-08
5605	chr3:49323951-49324091	USP4	Hs.77500	141	3.012	1.10E-12
111920	chr1:84909448-84909521	SSX2IP	Hs.22587	74	4.774	4.60E-08
113636	chr5:102546834-102547007	KIAA0433	Hs.212046	174	20.099	1.50E-41
55588	chr3:150226929-150226979	GYG	Hs.477892	51	6.971	9.70E-13
90905	chr16:2487712-2487729	KIAA1171	Hs.353087	18	3.712	1.20E-04
113638	chr5:102549920-102550039	KIAA0433	Hs.212046	120	6.099	9.20E-24
26589	chr4:177324269-177324289	WDR17	Hs.532056	21	3.657	8.20E-08
32121	chr10:75249296-75249409	CAMK2G	Hs.523045	114	10.23	3.40E-05
119206	chr16:24486614-24486715	RBBP6	Hs.188553	102	5.542	1.80E-09
117065	chr10:6308161-6308334	PFKFB3	Hs.195471	174	4.617	6.80E-13
81108	chr8:16992426-16992464	EFHA2	Hs.403594	39	12.423	8.10E-10
68001	chr14:69924940-69925076	SYNJ2BP	Hs.443661	137	3.484	4.00E-13
122626	chr1:116950637-116950696	IGSF3	Hs.171057	60	14.339	5.70E-18
48232	chr1:218258091-218258111	EPRS	Hs.497788	21	15.923	2.70E-11
35084	chr4:53939997-53940041	FIP1L1	Hs.518760	45	3.274	3.40E-04
87118	chr5:39161857-39161994	FYB	Hs.370503	138	5.226	6.20E-16
87117	chr5:39161864-39161994	FYB	Hs.370503	131	4.88	3.80E-13
37387	chr1:149373060-149373155	SEMA6C	Hs.516316	96	4.595	2.10E-10
116198	chr10:35530385-35530420	CREM	Hs.200250	36	20.774	3.80E-09
5803	chr1:94696751-94696787	ABCD3	Hs.76781	37	7.021	2.70E-07
60727	chr19:35153940-35153974	C19orf2	Hs.466391	35	4.551	2.60E-06
115600	chr1:36682192-36682221	NOR1	Hs.202207	30	5.239	1.10E-09
55619	chr3:147278339-147278401	PLOD2	Hs.477866	63	10.264	6.90E-13
40487	chr16:31105624-31105658	FUS	Hs.513522	35	7.531	1.10E-13
47060	chr10:103543660-103543745	MGEA5	Hs.500842	86	9.445	9.10E-19
49783	chr9:102148797-102148981	TEX10	Hs.494648	185	7.858	1.60E-29
71038	chr3:47024019-47024191	KIAA0540	Hs.437043	173	9.344	4.00E-26

4902	chr19:4369017-4369073	CHAF1A	Hs.79018	57	3.603	5.20E-06
125960	chr12:119273530-119273586	MSI1	Hs.158311	57	7.127	4.60E-13
83039	chr16:4821981-4822223	N-PAC	Hs.387255	243	3.974	4.90E-20
61219	chr19:1204953-1205081	MIDN	Hs.465529	129	9.876	1.40E-26
34651	chr5:133508387-133508459	TCF7	Hs.519580	73	4.54	4.60E-13
123724	chr9:23683446-23683484	ELAVL2	Hs.166109	39	3.228	1.20E-09
70996	chr3:47008969-47009049	KIAA0540	Hs.437043	81	4.724	8.00E-12
70529	chrX:119303508-119303579	FLJ20716	Hs.437563	72	6.467	3.60E-05
107248	chr17:7751644-7751745	CHD3	Hs.25601	102	4.252	2.50E-09
52383	chr6:131242977-131243039	EPB41L2	Hs.486470	63	3.348	2.00E-07
8266	chr15:46230589-46230660	MYEF2	Hs.6638	72	5.539	3.80E-07
44164	chr3:170983959-170984042	MYNN	Hs.507025	84	3.359	1.60E-10
48994	chrX:53325471-53325566	IQSEC2	Hs.496138	96	21.006	7.80E-10
125577	chr12:55773457-55773648	NAB2	Hs.159223	192	3.792	4.40E-15
9278	chr5:145611467-145611631	RBM27	Hs.61441	165	4.72	1.80E-16
62335	chr17:35444695-35444751	THRAP4	Hs.462983	57	12.069	8.60E-10
23639	chr2:152033234-152033311	RIF1	Hs.536537	78	24.602	2.80E-12
2080	chr2:96578785-96578923	ARID5A	Hs.920	139	15.87	1.00E-14
123680	chr6:33490990-33491084	PHF1	Hs.166204	95	4.545	1.10E-09
117271	chr7:150448007-150448165	CENTG3	Hs.195048	159	3.589	9.00E-17
117278	chr7:150467999-150468136	CENTG3	Hs.195048	138	3.706	4.30E-15
61569	chr18:9194474-9194542	ANKRD12	Hs.464585	69	5.755	3.80E-09
122591	chr1:37734895-37734924	FLJ11730	Hs.17118	30	6.07	4.00E-08
118692	chr3:52563780-52563935	PB1	Hs.189920	156	3.743	1.40E-15
108138	chr20:30845003-30845062	DNMT3B	Hs.251673	60	4.082	1.30E-04
123190	chr1:199218807-199218845	KIF21B	Hs.169182	39	6.411	6.40E-09
37371	chr2:96636434-96636511	FLJ10081	Hs.516341	78	10.168	6.40E-18
61562	chr18:9229475-9229554	ANKRD12	Hs.464585	80	4.645	2.70E-06
42537	chr11:118686269-118686386	MCAM	Hs.511397	118	3.384	1.30E-16
118691	chr3:52567305-52567469	PB1	Hs.189920	165	4.916	2.50E-26
102892	chr20:62325088-62325156	MYT1	Hs.279562	69	5.537	1.20E-12
82899	chr17:37306399-37306428	ACLY	Hs.387567	30	18.344	4.20E-07
93790	chr1:198275409-198275546	NR5A2	Hs.33446	138	4.112	3.20E-13
120489	chr1:203651983-203652047	LEMD1	Hs.181245	65	17.532	1.90E-05
125688	chr12:47747018-47747085	RHEBL1	Hs.159013	68	3.948	2.90E-07
79698	chr5:38985215-38985286	AVO3	Hs.407926	72	22.99	1.40E-14
122950	chr1:39802095-39802181	PABPC4	Hs.169900	87	36.236	3.20E-15
106912	chr2:36641432-36641512	FEZ2	Hs.258563	81	4.51	2.80E-13
43343	chr14:38859883-38860011	CTAGE5	Hs.509200	129	3.022	5.10E-14
69749	chrX:70290052-70290111	NLGN3	Hs.438877	60	6.706	1.50E-18
66405	chr11:73044496-73044600	PLEKHB1	Hs.445489	105	4.019	3.70E-08
66133	chr1:208479461-208479576	SERTAD4	Hs.445835	116	4.646	3.00E-11
511	chr8:1815621-1815737	ARHGEF10	Hs.98594	117	3.475	4.10E-19
29538	chr4:52630744-52630839	LOC132671	Hs.527090	96	3.67	1.40E-12
54130	chr5:39424164-39424226	DAB2	Hs.481980	63	4.812	2.00E-13
69183	chr1:6073910-6073954	KCNAB2	Hs.440497	45	5.434	1.30E-11
60943	chr19:10650289-10650380	ILF3	Hs.465885	92	7.039	1.90E-20
105633	chr5:138678263-138678324	MATR3	Hs.268939	62	105.566	1.30E-11
54709	chr4:85867038-85867088	WDFY3	Hs.480116	51	4.351	2.40E-11
71539	chr6:30716291-30716359	C6orf134	Hs.436521	69	3.034	2.80E-04
47667	chrX:99545178-99545318	PCDH19	Hs.4993	141	4.83	3.80E-10

95002	chr12:52183078-52183180	TARBP2	Hs.326	103	4.181	2.30E-11
105631	chr5:138671747-138671915	MATR3	Hs.268939	169	40.8	6.20E-14
94529	chr3:50187533-50187625	SEMA3F	Hs.32981	93	4.325	7.70E-11
9140	chr7:36434441-36434494	ANLN	Hs.62180	54	5.693	2.20E-10
49885	chr19:19606316-19606512	GMIP	Hs.49427	197	3.543	5.70E-15
110335	chr12:54852988-54853080	SMARCC2	Hs.236030	93	5.389	2.70E-17
3495	chr8:67228836-67229123	TRIM55	Hs.85524	288	3.48	5.60E-10
3496	chr8:67230412-67230499	TRIM55	Hs.85524	88	4.09	3.60E-12
22316	chr14:103265883-103265936	ZFYVE21	Hs.549192	54	3.133	1.10E-07
442	chr2:8805468-8805581	KIDINS220	Hs.9873	114	12.932	2.50E-20
34733	chr5:98232618-98232881	CHD1	Hs.519474	264	8.108	4.20E-21
110347	chr12:54844354-54844419	SMARCC2	Hs.236030	66	3.873	3.10E-09
443	chr2:8804726-8804782	KIDINS220	Hs.9873	57	5.733	4.50E-11
30611	chr12:121397875-121397979	RSN	Hs.524809	105	20.469	1.30E-23
75871	chr14:96413592-96413663	VRK1	Hs.422662	72	3.665	7.00E-07
100995	chr10:124177782-124177822	PLEKHA1	Hs.287830	41	5.978	1.10E-10
114649	chr4:178594424-178594499	AGA	Hs.207776	76	3.039	4.30E-11
30610	chr12:121401611-121401643	RSN	Hs.524809	33	4.122	1.60E-06
64300	chr9:139469512-139469580	NELF	Hs.455336	69	3.582	8.60E-07
8430	chr11:13333349-13333419	ARNTL	Hs.65734	71	5.801	2.40E-05
64299	chr9:139469512-139469604	NELF	Hs.455336	93	3.687	8.10E-06
119723	chr1:28226887-28227024	EYA3	Hs.185774	138	5.104	1.40E-21
58902	chr2:173932229-173932465	CDCA7	Hs.470654	237	3.926	7.40E-14
8431	chr11:13333356-13333419	ARNTL	Hs.65734	64	5.124	3.70E-05
37315	chr2:130635229-130635315	FLJ20297	Hs.516450	87	4.552	2.20E-10
38193	chr19:50979739-50979813	DMWD	Hs.515474	75	40.956	9.60E-16
39686	chr17:24930106-24930132	GIT1	Hs.514051	27	6.126	2.70E-04
105065	chr12:48813012-48813114	LASS5	Hs.270525	103	4.303	1.10E-08
75131	chr4:77287656-77287817	NUP54	Hs.430435	162	3.078	1.20E-10
115749	chr11:33326288-33326350	HIPK3	Hs.201918	63	14.649	2.50E-19
65087	chr17:33957521-33957643	SNIP	Hs.448872	123	3.919	8.80E-17
86312	chr9:92669234-92669302	SYK	Hs.371720	69	4.676	7.20E-16
115087	chr1:170813302-170813322	C1orf9	Hs.204559	21	23.804	6.60E-10
108070	chr7:73098454-73098483	ELN	Hs.252418	30	4.279	1.50E-04
72576	chr10:28924668-28924976	WAC	Hs.435610	309	3.344	2.90E-26
4245	chr5:36244484-36244549	FLJ30596	Hs.81907	66	12.263	1.10E-08
95943	chr14:26011366-26011437	NOVA1	Hs.31588	72	6.702	5.40E-08
39724	chr17:24235369-24235459	FLOT2	Hs.514038	91	4.909	1.40E-16
77260	chr16:23242376-23242400	SCNN1B	Hs.414614	25	4.009	2.00E-04
50369	chr1:160829146-160829196	UAP1	Hs.492859	51	12.261	1.10E-09
90162	chr14:103034592-103034618	MARK3	Hs.35828	27	39.123	7.70E-06
39722	chr17:24237001-24237091	FLOT2	Hs.514038	91	7.772	2.60E-21
35082	chr4:53952354-53952422	FIP1L1	Hs.518760	69	8.682	3.30E-10
18194	chr16:67712457-67712574	DERPC	Hs.560414	118	3.99	1.90E-05
29576	chr4:3400083-3400236	RGS12	Hs.527061	154	7.565	3.80E-20
60946	chr19:10652022-10652119	ILF3	Hs.465885	98	5.536	3.40E-18

B, Mouse (262 exons)

From total dataset, the exons with RSPR >3.0 and P_RSPR<0.001 are selected.

EXON_ID	LOCATION IN mm9	GENE SYMBOL	CLUSTER_ID	EXON LENGTH	RSPR	P_{RSPR}
51949	chr8:3600358-3600405	2310057J16Ri	Mm.28248	48	7.098	7.50E-11
40222	chr17:24738334-24738402	Tsc2	Mm.30435	69	7.265	2.40E-21
28609	chr16:17635575-17635661	4122402O22Ri	Mm.34659	87	4.559	1.00E-09
17809	chr17:44795468-44795641	Runx2	Mm.386756	174	19.477	2.60E-15
62468	chr15:66429227-66429316	Phf20l1	Mm.267473	90	7.417	6.80E-12
81062	chr9:70431739-70431919	5730555F13Ri	Mm.22379	181	4.163	1.70E-14
73865	chr1:164605736-164605972	1810043M20Ri	Mm.245446	237	5.083	3.80E-31
84252	chr15:58778881-58779075	Mtss1	Mm.215481	195	5.459	3.40E-14
63575	chr1:173316291-173316365	Pvrl4	Mm.263414	75	4.714	2.80E-08
48715	chr3:138646686-138646832	Rap1gds1	Mm.28893	147	4.261	2.40E-17
94117	chr3:58843088-58843120	P2ry14	Mm.17803	33	3.692	7.90E-05
86444	chrX:45309387-45309410	9530014D17Ri	Mm.210343	24	5.975	1.20E-08
81053	chr9:70421492-70421732	5730555F13Ri	Mm.22379	241	4.084	2.30E-15
76320	chr11:51086580-51086670	Clk4	Mm.239354	91	14.009	6.00E-18
71624	chr5:106317453-106317858	Zfp326	Mm.248876	406	28.704	3.80E-36
15386	chr17:29339800-29339850	Cpne5	Mm.39905	51	5.953	1.10E-08
63012	chr5:54501355-54501378	Stim2	Mm.265985	24	3.815	6.10E-04
10599	chr1:12840841-12840874	Sulf1	Mm.45563	34	5.759	6.50E-10
96863	chr5:42183036-42183176	AF013969	Mm.158726	141	4.887	1.20E-07
24869	chr2:91004120-91004179	Madd	Mm.36410	60	5.268	8.20E-16
86728	chr2:73661258-73661363	Atf2	Mm.209903	106	3.856	8.90E-22
98975	chr3:97513405-97513506	None	Mm.150236	102	3.963	3.10E-09
62125	chr11:69717179-69717221	0610025P10Ri	Mm.268347	43	6.783	3.40E-16
52138	chr17:46157940-46158071	Vegfa	Mm.282184	132	5.749	1.30E-18
62542	chr14:52530761-52530802	Ndrg2	Mm.26722	42	3.191	2.20E-08
31013	chr11:29397343-29397426	A430106J12Ri	Mm.338284	84	7.02	2.20E-18
75932	chr8:14942610-14942726	Arhgef10	Mm.240298	117	3.024	1.90E-22
24875	chr2:91000474-91000527	Madd	Mm.36410	54	12.269	9.40E-16
84521	chr18:35732421-35732589	Matr3	Mm.215034	169	5.852	6.70E-23
36568	chr7:138053102-138053172	AA960558	Mm.323554	71	3.391	2.10E-09
78626	chr3:30847624-30847722	Phc3	Mm.233173	99	4.58	4.30E-15
74716	chr12:71123268-71123328	Nin	Mm.244047	61	3.143	4.80E-09
83018	chr16:16318538-16318570	Dnm1l	Mm.218820	33	20.911	1.50E-06
83017	chr16:16318857-16318934	Dnm1l	Mm.218820	78	5.683	2.40E-05
71335	chr2:71071706-71071765	Dncic2	Mm.249479	60	3.876	2.30E-15
54323	chr4:71799996-71800097	Tle1	Mm.278444	102	3.17	2.50E-07
55825	chr2:23397039-23397135	4921517N04Ri	Mm.276415	97	6.007	7.20E-06
24893	chr2:90980714-90980783	Madd	Mm.36410	70	8.425	7.30E-21
96684	chrX:157692962-157693113	Scml2	Mm.159173	152	3.541	5.10E-10
47582	chr10:60239933-60239965	Unc5b	Mm.290433	33	6.69	2.50E-08
55179	chr6:30377709-30377795	2410127E18Ri	Mm.277308	87	6.977	3.90E-13
26298	chr7:86253001-86253114	Agc1	Mm.358571	114	5.745	9.90E-22
31357	chr10:126527198-126527257	None	Mm.336761	60	3.806	3.20E-11
66788	chr3:33963896-33963976	Fxr1h	Mm.259021	81	5.996	2.00E-18
15185	chr4:151759833-151759947	Chd5	Mm.40192	115	4.851	5.30E-14
98733	chr11:69644065-69644115	Nlgn2	Mm.151293	51	30.393	5.70E-18
42162	chr12:107309780-107309851	Vrk1	Mm.2981	72	5.181	1.20E-05
3976	chr10:31057991-31058051	Tpd52l1	Mm.7821	61	3.313	1.40E-07
77031	chr17:78786331-78786411	Fez2	Mm.236835	81	4.816	4.30E-15
88640	chr2:29884164-29884226	Spna2	Mm.204969	63	4.117	4.80E-14
67196	chr4:32081849-32081929	Map3k7	Mm.258589	81	3.82	2.90E-09
2022	chr6:4640469-4640495	Sgce	Mm.8739	27	5.033	5.20E-07
36630	chr15:76560794-76560886	D15Wsu169e	Mm.322931	93	3.998	1.00E-16
104247	chrX:98498411-98498470	Nlgn3	Mm.121508	60	7.182	2.50E-22
60341	chr11:62173263-62173436	Ncor1	Mm.271814	174	3.377	1.40E-21

53865	chr14:31927035-31927190	2610016F04Ri	Mm.27913	156	4.358	1.10E-18
53864	chr14:31923601-31923765	2610016F04Ri	Mm.27913	165	4.343	1.10E-22
95131	chr1:163775691-163775711	AI848100	Mm.170002	21	30.965	4.70E-09
26526	chr2:84452689-84452706	Catns	Mm.35738	18	5.774	1.30E-08
45935	chr17:80601509-80601544	Sfrs7	Mm.292016	36	4.471	9.10E-08
84760	chr6:34974128-34974340	Cnot4	Mm.214525	213	5.382	2.00E-21
2015	chr6:4680006-4680113	Sgce	Mm.8739	108	9.024	2.10E-12
8170	chr17:23943899-23944169	Srrm2	Mm.5222	271	5.826	1.10E-13
104308	chr5:73357043-73357120	5033405K12Ri	Mm.121285	78	13.488	1.70E-09
47569	chr14:58468982-58469017	5033406L14Ri	Mm.29044	36	3.358	1.10E-05
23086	chr13:52720077-52720145	Syk	Mm.375031	69	5.376	1.90E-15
97375	chr3:108158390-108158488	Sort1	Mm.157119	99	9.559	4.00E-13
77889	chr14:99452737-99452782	6720463M24Ri	Mm.23503	46	5.149	1.40E-10
14184	chr15:90806902-90806940	Kif21a	Mm.41379	39	15.404	2.30E-11
75613	chr5:124085183-124085287	Rsn	Mm.241109	105	19.264	1.40E-23
13373	chr4:129758679-129758723	Col16a1	Mm.41860	45	7.256	1.30E-13
91944	chr13:55577997-55578134	Dbn1	Mm.19016	138	5.96	6.10E-19
61052	chr1:5091151-5091204	Atp6v1h	Mm.27082	54	3.084	2.90E-04
89693	chr3:30511391-30511474	Mynn	Mm.200378	84	5.551	2.40E-12
1794	chr1:183865791-183865847	Enah	Mm.87759	57	8.966	7.80E-09
102861	chr9:107593687-107593779	Sema3f	Mm.12903	93	3.654	1.20E-13
82768	chr5:120125252-120125311	Tbx3	Mm.219139	60	5.568	6.10E-08
29569	chr13:46856032-46856136	Kif13a	Mm.342703	105	4.1	1.90E-10
73748	chr14:70625735-70625761	Ppp3cc	Mm.245742	27	7.126	6.30E-05
33535	chr4:140676239-140676288	D4Ertd22e	Mm.332006	50	3.312	3.70E-10
51228	chr6:83249420-83249504	C330016K18Ri	Mm.284370	85	3.4	2.10E-04
70733	chr12:25725321-25725434	C330002I19Ri	Mm.250641	114	11.096	1.10E-23
17378	chr7:134000740-134000856	AI225782	Mm.386867	117	5.949	3.40E-14
26820	chr13:107759784-107759897	Kif2a	Mm.355686	114	3.933	7.90E-10
27802	chr1:137360492-137360515	Nav1	Mm.34977	24	5.421	2.10E-05
58172	chr16:29588909-29588962	Opa1	Mm.274285	54	3.261	3.10E-06
14944	chr9:80151080-80151106	Myo6	Mm.4040	27	5.063	1.80E-07
98401	chr12:86401213-86401264	Ylpm1	Mm.153183	52	4.124	1.10E-11
69428	chr11:119969781-119969867	1810073N04Ri	Mm.253403	87	3.394	1.20E-04
33940	chr3:101969696-101969734	Vangl1	Mm.331266	39	3.517	7.80E-06
382	chr4:63645494-63645766	Tnc	Mm.980	273	3.374	3.50E-37
82811	chr7:117078996-117079067	Rab6ip1	Mm.21904	72	4.216	6.50E-14
31128	chr8:73860775-73860864	Myo9b	Mm.33779	90	3.556	4.90E-20
64223	chr5:134748394-134748456	Gtf2i	Mm.261570	63	4.843	8.20E-07
11736	chr6:55441221-55441304	Adcyap1r1	Mm.44245	84	4.858	1.20E-16
31954	chr9:50884427-50884450	E030012M19Ri	Mm.335152	24	6.966	3.30E-05
14310	chr7:26558118-26558144	Axl	Mm.4128	27	11.938	4.50E-04
80487	chr11:102965214-102965334	Acbd4	Mm.225881	121	43.135	9.10E-11
65106	chr2:60085545-60085569	Axot	Mm.260635	25	5.646	3.10E-06
25222	chr9:86400478-86400507	B130005I07Ri	Mm.360478	30	4.429	1.80E-12
92535	chr5:100804980-100805072	Sec31l1	Mm.18634	93	15.324	2.00E-20
92422	chr1:134901231-134901298	Mdm4	Mm.18706	68	6.887	1.80E-09
82046	chr1:192657885-192657977	Rps6kc1	Mm.220912	93	7.964	2.50E-25
11551	chr3:86634746-86634793	6330415M09Ri	Mm.44490	48	7.231	8.60E-07
14298	chr6:50126876-50126917	Mpp6	Mm.41288	42	4.001	5.20E-05
27226	chr17:35042555-35042656	Bat8	Mm.35345	102	7.275	7.80E-22
23542	chr17:57396283-57396450	Trip10	Mm.37368	168	4.428	2.80E-14
45753	chr18:34619011-34619076	2610024E20Ri	Mm.292258	66	6.797	1.80E-11
69296	chr2:26244436-26244585	C330016H24Ri	Mm.253771	150	3.818	2.00E-30
36021	chr16:26965662-26965836	None	Mm.326909	175	4.692	4.60E-16
41440	chr4:131519844-131519885	Epb4.1	Mm.30038	42	3.684	3.00E-08
73295	chrX:69509082-69509164	Gabre	Mm.246514	83	3.38	6.00E-11
26112	chr15:99135597-99135624	2610317D23Ri	Mm.358668	28	4.219	6.10E-05
90793	chr5:115187357-115187446	Git2	Mm.195632	90	4.261	1.00E-17
54122	chr2:25149958-25150068	Grin1	Mm.278672	111	9.294	9.20E-22

12620	chr9:58732277-58732435	Neo1	Mm.42249	159	4.857	4.70E-35
57796	chr3:94659965-94660123	Pogz	Mm.274787	159	4.062	9.50E-19
53143	chr9:113868494-113868601	Ubp1	Mm.28052	108	4.05	4.50E-13
4957	chr5:43629566-43629655	Cpeb2	Mm.7233	90	7.458	1.30E-11
33637	chr10:75734121-75734150	Dip2	Mm.33183	30	3.367	5.30E-08
90583	chr3:95590243-95590374	4930535B03Ri	Mm.196275	132	11.636	2.70E-19
12615	chr9:58749890-58749922	Neo1	Mm.42249	33	4.028	5.80E-11
97665	chr2:34555346-34555426	4432404J10Ri	Mm.156452	81	4.061	7.80E-15
69827	chr8:71348089-71348166	4921521J11Ri	Mm.252421	78	5.553	5.70E-08
54934	chr8:83300060-83300149	Gab1	Mm.277409	90	4.169	4.90E-18
99813	chr10:79616336-79616464	Midn	Mm.143813	129	10.396	3.60E-29
35885	chrX:33879515-33879670	A830039N02Ri	Mm.327654	156	5.675	3.50E-09
85320	chr18:62674449-62675198	Fbxo38	Mm.21295	750	4.865	3.10E-35
77558	chr7:133293018-133293051	Xpo6	Mm.235663	34	16.368	1.20E-04
53210	chr14:21286680-21286745	Anxa7	Mm.280231	66	3.964	8.20E-07
68233	chr19:6368364-6368439	Zfp162	Mm.256422	76	23.574	1.10E-29
69828	chr8:71348951-71349028	4921521J11Ri	Mm.252421	78	10.893	2.40E-23
27755	chr7:149699570-149699610	Tnnt3	Mm.350054	41	4.277	1.40E-04
102935	chr11:104166526-104166723	Mapt	Mm.1287	198	8.718	2.40E-12
41850	chr4:44043087-44043140	Clta	Mm.298875	54	19.027	9.00E-09
35969	chr16:32841623-32841667	1700021K19Ri	Mm.327319	45	3.165	6.40E-08
34386	chr2:124486441-124486497	Sema6d	Mm.330536	57	25.117	1.80E-15
86154	chr8:46868897-46868980	9430041O17Ri	Mm.211096	84	4.259	7.70E-10
10526	chr18:34770064-34770282	Brd8	Mm.45602	219	10.628	7.30E-22
34388	chr2:124487940-124488107	Sema6d	Mm.330536	168	5.764	2.30E-25
86395	chr8:4201934-4201966	B130050I23Ri	Mm.210436	33	3.453	3.00E-07
11547	chr3:86592544-86592556	6330415M09Ri	Mm.44490	13	12.01	6.30E-04
79959	chr1:167297577-167297636	Iqwd1	Mm.227605	60	4.664	2.80E-10
2801	chr7:137888041-137888130	Tacc2	Mm.86322	90	3.036	8.40E-10
17433	chr1:58591329-58591496	BC049806	Mm.386850	168	8.734	5.60E-20
2792	chr7:137875045-137875080	Tacc2	Mm.86322	36	3.511	7.30E-07
29914	chr11:77629358-77629393	Myo18a	Mm.341248	36	5.736	5.80E-07
92540	chr5:100798615-100798653	Sec31l1	Mm.18634	39	3.434	1.30E-04
9280	chr11:23522436-23522529	0610010F05Ri	Mm.48031	94	5.422	3.80E-11
43882	chrX:71478306-71478329	Flna	Mm.295533	24	7.053	2.80E-07
34392	chr1:175279153-175279254	Igsf4b	Mm.330524	102	6.309	2.50E-13
84522	chr18:35737212-35737273	Matr3	Mm.215034	62	3.176	5.80E-10
41439	chr4:131520046-131520102	Epb4.1	Mm.30038	57	3.945	8.20E-12
97782	chr6:51414427-51414546	Hnrpa2b1	Mm.155896	120	4.929	5.70E-13
41441	chr4:131518839-131518901	Epb4.1	Mm.30038	63	8.221	2.90E-13
73584	chr7:105793677-105793718	2210018M11Ri	Mm.246010	42	11.357	2.80E-06
54741	chr18:32062236-32062331	Wdr33	Mm.277705	96	13.868	1.30E-09
69327	chr1:40546446-40546585	Il18r1	Mm.253664	140	4.237	8.10E-08
95025	chr15:6594762-6594899	Fyb	Mm.170905	138	4.619	3.00E-08
12343	chr15:74407825-74407923	Bai1	Mm.43133	99	3.559	7.10E-06
2573	chr12:83034518-83034702	Pcnx	Mm.86584	185	4.964	9.50E-15
82023	chr2:156573759-156573838	Dlgap4	Mm.22094	80	4.125	3.40E-05
73107	chrX:82951577-82951663	Gyk	Mm.246682	87	6.395	9.70E-17
773	chr11:28296046-28296165	E030025D05Ri	Mm.93759	120	11.625	3.40E-19
774	chr11:28292854-28292952	E030025D05Ri	Mm.93759	99	34.409	7.90E-18
66568	chr9:107662107-107662180	Rbm5	Mm.259197	74	28.299	2.90E-22
71498	chr6:91209624-91209764	Fbln2	Mm.249146	141	3.188	3.60E-20
5693	chrX:130607217-130607303	Cstf2	Mm.67938	87	11.731	1.50E-13
61473	chr2:126739036-126739089	2010106G01Ri	Mm.269928	54	5.409	2.80E-11
27006	chr14:66975106-66975186	Stmn4	Mm.35474	81	6.11	6.40E-10
93963	chr2:27123671-27123757	Vav2	Mm.179011	87	36.48	5.60E-18
79786	chr13:55216841-55216985	Zfp346	Mm.22795	145	3.021	1.20E-06
40735	chr9:111116317-111116418	Lrrfip2	Mm.302287	102	3.153	2.20E-10
12423	chr11:70422091-70422201	Map4k6	Mm.42967	111	6.032	1.50E-22
31186	chr2:180713117-180713146	Arfgap1	Mm.33765	30	6.075	1.20E-05

38585	chrX:54044416-54044615	Fhl1	Mm.3126	200	46.935	4.40E-10
69068	chr11:54485157-54485180	Rapgef6	Mm.254404	24	5.413	5.10E-10
86933	chr9:21199815-21199912	Ilf3	Mm.20935	98	5.764	2.80E-18
36138	chr14:47403501-47403548	Cnih	Mm.3261	48	4.58	9.30E-09
45811	chr2:121751297-121751464	D130060C09Ri	Mm.292109	168	4.505	4.60E-17
61304	chr5:89027461-89027514	D5Bwg0860e	Mm.270469	54	7.739	2.90E-07
91456	chr18:55106207-55106462	Zfp608	Mm.192984	256	4.288	1.00E-25
75612	chr5:124088965-124088997	Rsn	Mm.241109	33	3.042	1.50E-06
21634	chr3:87522151-87522180	Arhgef11	Mm.379340	30	11.674	1.70E-09
23796	chr8:125013455-125013526	2310061F22Ri	Mm.37324	72	3.403	1.60E-08
11297	chr7:130141110-130141211	Rbbp6	Mm.4480	102	3.959	5.10E-09
29653	chr12:121254606-121254679	None	Mm.342227	74	11.189	4.00E-24
68112	chr5:76969772-76969792	Sec3l1	Mm.256624	21	6.729	4.80E-06
68944	chr11:86514563-86514583	Cltc	Mm.254588	21	8.615	8.90E-13
22995	chr7:107255073-107255205	Pold3	Mm.37562	133	3.741	4.10E-15
98608	chr10:80244888-80244929	Dot1l	Mm.152371	42	3.535	6.70E-08
48932	chr5:130065344-130065496	Sfrs8	Mm.288714	153	5.173	5.00E-25
67768	chrX:17824693-17824848	Utx	Mm.257498	156	5.697	6.90E-08
28186	chr2:29824296-29824346	None	Mm.347812	51	4.188	6.80E-08
63344	chr5:36947160-36947257	D5Ertd579e	Mm.26480	98	3.299	2.20E-15
46881	chr11:49977664-49977714	2700008N14Ri	Mm.290975	51	4.854	5.50E-09
95201	chr7:119190441-119190500	Usp47	Mm.16974	60	5.474	6.80E-12
65076	chr6:29151888-29151904	Impdh1	Mm.260707	17	14.606	4.40E-07
63808	chr5:138049845-138049919	Pcolce	Mm.262345	75	3.021	6.10E-05
43292	chr2:35576781-35576933	Dab2ip	Mm.29629	153	4.31	1.00E-11
67760	chrX:17810986-17811040	Utx	Mm.257498	55	3.021	3.50E-09
40005	chr5:31377844-31377894	Cad	Mm.305535	51	3.633	5.30E-10
29422	chr1:36374049-36374169	Arid5a	Mm.34316	121	7.193	1.90E-16
62124	chr11:69717156-69717221	0610025P10Ri	Mm.268347	66	5.003	5.30E-18
65105	chr2:60083269-60083317	Axot	Mm.260635	49	3.847	6.70E-09
102936	chr11:104166627-104166723	Mapt	Mm.1287	97	8.741	8.60E-15
10528	chr18:34768072-34768281	Brd8	Mm.45602	210	4.217	3.50E-29
77781	chr18:54108299-54108322	Csnk1g3	Mm.235240	24	4.75	2.90E-04
3687	chr9:92496823-92496885	Plod2	Mm.79983	63	10.046	1.60E-12
11622	chr5:44119510-44119530	5730509K17Ri	Mm.44434	21	3.351	2.80E-06
27444	chr10:115944097-115944244	Cnot2	Mm.351553	148	3.306	3.50E-12
39047	chr12:34049250-34049555	None	Mm.310519	306	7.307	8.10E-16
26961	chr15:89126383-89126460	Sbf1	Mm.35483	78	4.888	3.10E-14
68973	chr2:51972188-51972265	Rif1	Mm.254530	78	36.072	1.20E-13
80698	chr10:109197543-109197584	Nav3	Mm.225050	42	5.185	5.10E-14
6365	chr1:82876583-82876702	Hrb	Mm.6461	120	4.824	3.70E-17
59274	chr1:89671388-89671444	Apg16l	Mm.272972	57	8.626	1.50E-14
83263	chr4:137175838-137175889	Usp48	Mm.218478	52	9.917	1.80E-11
10717	chr4:154843571-154843612	2310043I08Ri	Mm.45401	42	4.129	4.00E-10
10716	chr4:154843553-154843612	2310043I08Ri	Mm.45401	60	6.959	1.20E-11
104058	chr19:45836675-45836760	Mgea5	Mm.122725	86	8.799	3.30E-24
61444	chr8:72436063-72436137	BC031407	Mm.270044	75	6.7	2.00E-20
59276	chr1:89671656-89671703	Apg16l	Mm.272972	48	6.514	1.10E-11
90237	chr2:11401825-11401998	Pfkfb3	Mm.19669	174	4.474	1.10E-13
28356	chr15:76329562-76329645	Hsf1	Mm.347444	84	5.65	1.10E-05
98180	chr1:83262544-83262630	4930544G21Ri	Mm.154303	87	10.874	9.20E-21
8994	chr17:48538307-48538393	Nfya	Mm.4929	87	5.298	1.30E-26
90584	chr3:95590243-95590320	4930535B03Ri	Mm.196275	78	14.894	3.70E-19
16012	chr8:4240688-4240735	Map2k7	Mm.3906	48	10.102	1.20E-15
73084	chr6:47496748-47496829	Ezh2	Mm.246688	82	14.311	2.10E-12
77779	chr18:54096507-54096602	Csnk1g3	Mm.235240	96	6.99	1.40E-09
9553	chr15:96528353-96528468	Slc38a2	Mm.46754	116	3.478	1.60E-14
42993	chr18:14795901-14795993	None	Mm.296783	93	13.512	7.20E-25
88865	chr17:35279443-35279550	Bat3	Mm.203962	108	8.546	3.20E-25
103044	chr2:3389774-3389919	Suv39h2	Mm.128273	146	3.717	3.00E-14

16036	chr11:83178297-83178338	Ap2b1	Mm.39053	42	3.235	5.60E-13
29557	chr13:46875995-46876033	Kif13a	Mm.342703	39	7.976	9.10E-07
6497	chr3:20023008-20023058	Gyg1	Mm.6375	51	7.663	9.40E-12
14845	chr2:180826944-180826997	Kcnq2	Mm.40615	54	4.557	1.40E-08
94111	chr16:36721624-36721755	Ildr1	Mm.17807	132	10.094	3.90E-20
56728	chr15:6737650-6737721	4921505C17Ri	Mm.275811	72	23.248	7.60E-15
413	chr3:148490505-148490549	Lphn2	Mm.9776	45	4.942	2.30E-15
2028	chr12:9002446-9002478	Wdr35	Mm.87389	33	3.752	1.00E-06
29964	chr12:8740079-8740315	Pum2	Mm.341243	237	3.419	4.40E-32
421	chr3:148484244-148484372	Lphn2	Mm.9776	129	4.047	7.10E-23
425	chr3:148483415-148483432	Lphn2	Mm.9776	18	20.838	4.80E-11
424	chr3:148483937-148483989	Lphn2	Mm.9776	53	6.836	9.40E-10
18045	chr2:4569143-4569169	Prpf18	Mm.38529	27	12.677	4.40E-08
78945	chr2:163884402-163884549	Tomm34	Mm.23173	148	3.334	3.70E-16
61241	chr6:82880892-82880985	Sema4f	Mm.270543	94	3.537	1.60E-20
428	chr3:148512730-148512768	Lphn2	Mm.9776	39	5.814	7.00E-15
88854	chr17:35283702-35283848	Bat3	Mm.203962	147	5.917	8.50E-19
65308	chr7:20014455-20014534	Mark4	Mm.260504	80	5.439	2.20E-09
414	chr3:148490176-148490202	Lphn2	Mm.9776	27	8.665	2.70E-07
2895	chr7:133611464-133611563	Sh2bpsm1	Mm.8538	100	3.611	3.40E-11
87327	chr18:67672495-67672536	Spire1	Mm.208723	42	4.609	6.60E-09
68755	chr17:29121022-29121145	Stk38	Mm.255075	124	3.191	2.70E-21
102025	chr12:53013192-53013218	D930036F22Ri	Mm.131941	27	7.968	2.20E-10
62469	chr15:66429239-66429316	Phf20l1	Mm.267473	78	6.391	2.60E-10
73866	chr1:164604230-164604282	1810043M20Ri	Mm.245446	53	30.761	1.30E-12
19089	chr11:116701152-116701240	Ptdsr	Mm.383423	89	37.302	1.20E-16
81051	chr9:70421264-70421732	5730555F13Ri	Mm.22379	469	4.836	3.60E-15
