# Supplementary material for: Predicting Functional Alternative Splicing by Measuring RNA Selection Pressure from Multigenome Alignments
Source: PLoS Comput Biol. 2009 Dec 18;5(12):e1000608. doi: 10.1371/journal.pcbi.1000608 (PMC2784930; doi:10.1371/journal.pcbi.1000608)
Supplement: Table S3 — Confirmed functional alternative splicing in predicted dataset. RSPR predictions that were validated as functional AS events by published literature. (0.12 MB PDF) [file pcbi.1000608.s003.pdf]

Table S3. Confirmed functional alternative splicing in predicted dataset.

GENE	DESCRIPTION	EXON ID*	RSPR	P_RSPR	REFERENCES
GRIN1	Glutamate receptor, ionotropic, N-methyl D-aspartate 1	19712	8.92	7.72×10^{-25}	[1]
		19695	8.46	1.40×10^{-13}	
SYK	Spleen tyrosine kinase	86312	4.68	7.10×10^{-16}	[2]
DGKH	Diacylglycerol kinase, eta	18868	3.71	4.03×10^{-4}	[3]
WT1	Wilms tumor 1	79366	3.73	1.25×10^{-4}	[4]
LRP8 (*ApoER2*)	Low density lipoprotein receptor-related protein 8, apolipoprotein e receptor	102438	6.08	3.32×10^{-4}	[5]

**An exon is identified by EXON ID defined in ASAPII*

References:

1. Llansola M, Sanchez-Perez A, Cauli O, Felipo V (2005) Modulation of NMDA receptors in the cerebellum. 1. Properties of the NMDA receptor that modulate its function. Cerebellum 4: 154-161.
2. Wang L, Duke L, Zhang PS, Arlinghaus RB, Symmans WF, et al. (2003) Alternative splicing disrupts a nuclear localization signal in spleen tyrosine kinase that is required for invasion suppression in breast cancer. Cancer Res 63: 4724-4730.
3. Murakami T, Sakane F, Imai S, Houkin K, Kanoh H (2003) Identification and characterization of two splice variants of human diacylglycerol kinase eta. J Biol Chem 278: 34364-34372.
4. Richard DJ, Schumacher V, Royer-Pokora B, Roberts SG (2001) Par4 is a coactivator for a splice isoform-specific transcriptional activation domain in WT1. Genes Dev 15: 328-339.
5. Koch S, Strasser V, Hauser C, Fasching D, Brandes C, et al. (2002) A secreted soluble form of ApoE receptor 2 acts as a dominant-negative receptor and inhibits Reelin signaling. EMBO J 21: 5996-6004.
