# Supplementary material for: Predicting Functional Alternative Splicing by Measuring RNA Selection Pressure from Multigenome Alignments
Source: PLoS Comput Biol. 2009 Dec 18;5(12):e1000608. doi: 10.1371/journal.pcbi.1000608 (PMC2784930; doi:10.1371/journal.pcbi.1000608)
Supplement: Table S4 — RT-PCR Analysis of 20 High-RSPR AS Exons vs. 20 Low-RSPR AS Exons. No. 1–20 exons are with high RSPR (RSPR>3.0, P_RSPR<0.001), No. 21–40 exons are with low RSPR (RSPR<1.0, P_RSPR<0.001). Tissue-specific AS: T-AS, Non-Tissue-specific AS: NT-AS, Constitutive: Const; Solid arrow: Inclusion, Hollow arrow: Skipping, Dashed arrow: Non-specific amplification by PCR. (3.20 MB PDF) [file pcbi.1000608.s004.pdf]

Table S4. RT-PCR Analysis of 20 High-RSPR AS Exons vs. 20 Low-RSPR AS Exons.

No. 1~20 exons are with high RSPR (RSPR>3.0, P_RSPR<0.001), No. 21~40 exons are with low RSPR (RSPR<1.0, P_RSPR<0.001). Tissue-specific AS: T-AS, Non-Tissue-specific AS: NT-AS, Constitutive: Const; Solid arrow: Inclusion, Hollow arrow: Skipping, Dashed arrow: Non-specific amplification by PCR.

Id	Gene Symbol	Inclusion Size	Exclusion Size	Exon Length
1	PABPC4	299/251	212/164	87
	RSPR	$P_{\_RSPR}$	Location in hg18	T-AS
	36.2357	3E-15	chr1:39802095-39802181	
Left Primer	gggcctcgtccaactctt			
Right Primer	tgcagaggctgtatggca			

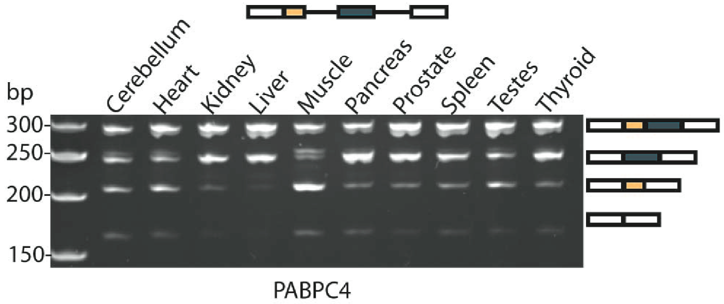

The target exon with RSPR annotated is in black.

Id	Gene Symbol	Inclusion Size	Exclusion Size	Exon Length
2	RIF1	271	193	78
	RSPR	$P_{\_RSPR}$	Location in hg18	T-AS
	24.602	2E-12	chr2:152033234-152033311	
Left Primer	accaagcaggattggcag			
Right Primer	ggtgccacacagttcacc			

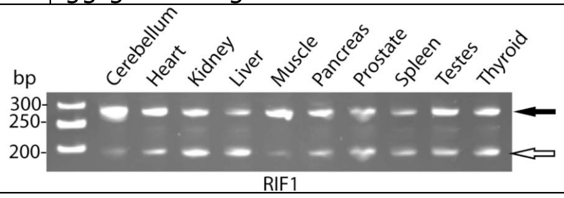

Id	Gene Symbol	Inclusion Size	Exclusion Size	Exon Length
3	UAP1	300	249	51
	RSPR	$P_{\_RSPR}$	Location in hg18	T-AS
	12.2613	1E-09	chr1:160829146-160829196	
Left Primer	gtcctcaatgcaggggg			
Right Primer	cgtggcaaacaaaacttgg			

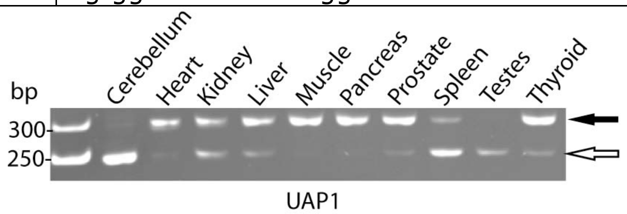

Id	Gene Symbol	Inclusion Size	Exclusion Size	Exon Length
4	BRD8	464	245	219
	RSPR	P_{RSPR}	Location in hg18	T-AS
	8.8782	1E-19	chr5:137532058-137532276	
Left Primer	agattctgcctccccagg			
Right Primer	aaagccgggaaagagtgg			

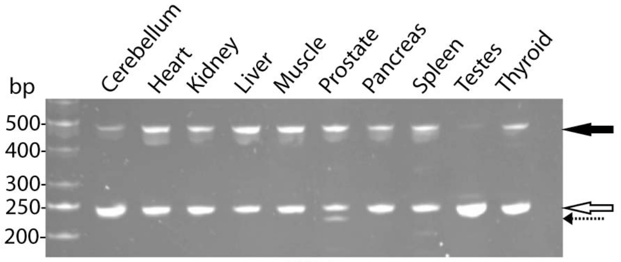

BRD8

Id	Gene Symbol	Inclusion Size	Exclusion Size	Exon Length
5	BAI2	222	123	99
	RSPR	P_{RSPR}	Location in hg18	NT-AS
	6.8373	4E-11	chr1:31973371-31973469	
Left Primer	caacaagctcatggcacg			
Right Primer	aggacggcagacatccag			

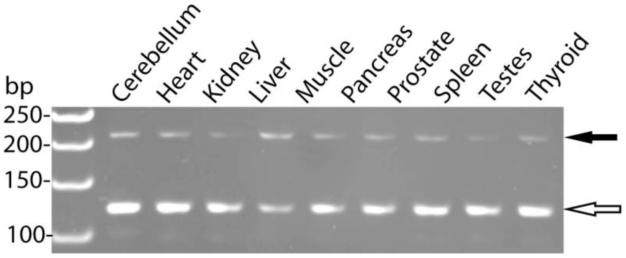

BAI2

Id	Gene Symbol	Inclusion Size	Exclusion Size	Exon Length
6	FLJ20716	243	171	72
	RSPR	P_{RSPR}	Location in hg18	T-AS
	6.4674	3E-05	chrX:119303508-119303579	
Left Primer	tgccatagttgacggggt			
Right Primer	cagaagcaggtgttgccc			

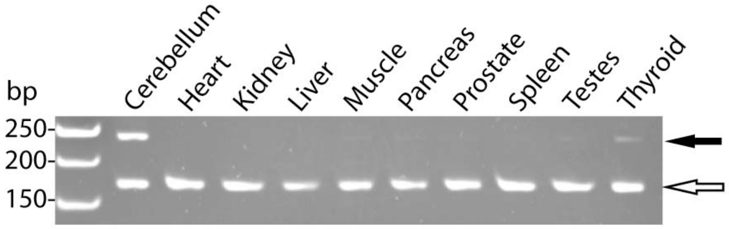

FLJ20716

Id	Gene Symbol	Inclusion Size	Exclusion Size	Exon Length
7	GIT1	163	136	27
	RSPR	P_RSPR	Location in hg18	T-AS
	6.1257	2E-04	chr17:24930106-24930132	
Left Primer		acctctgtggacgcaagc		
Right Primer		tcctcaaaaagccggttg		

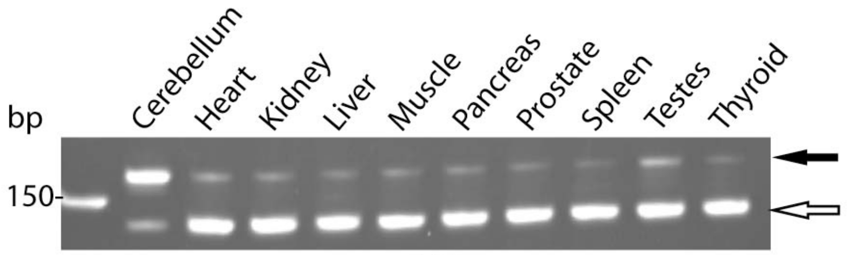

GIT1

Id	Gene Symbol	Inclusion Size	Exclusion Size	Exon Length
8	KIAA1841	289	195	94
	RSPR	P_RSPR	Location in hg18	T-AS
	5.6753	2E-11	chr2:61154097-61154190	
Left Primer		tccctaatggctgctgga		
Right Primer		gatctcgcgggcaagtaa		

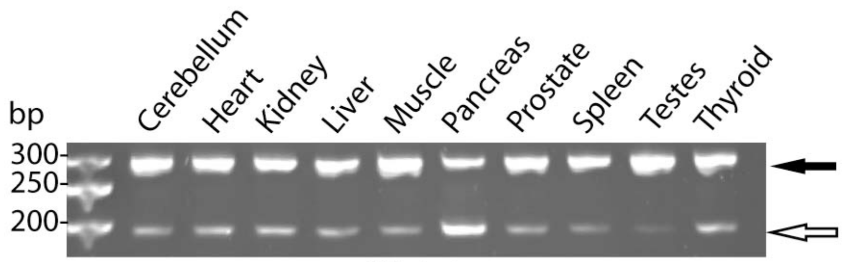

KIAA1841

Id	Gene Symbol	Inclusion Size	Exclusion Size	Exon Length
9	NOR1	216	186	30
	RSPR	P_RSPR	Location in hg18	T-AS
	5.2386	1E-09	chr1:36682192-36682221	
Left Primer		gcggggagatgctttaca		
Right Primer		gagccaggcgctcataga		

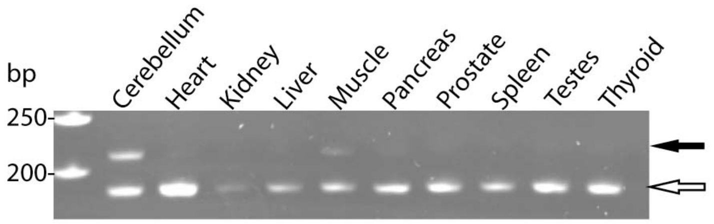

NOR1

Id	Gene Symbol	Inclusion Size	Exclusion Size	Exon Length
10	PCM1	364	247	117
	RSPR	P_RSPR	Location in hg18	NT-AS
	5.051	2E-18	chr8:17841831-17841947	
Left Primer		agctagttccatgcggga		
Right Primer		ctgccgtccctgtagagc		

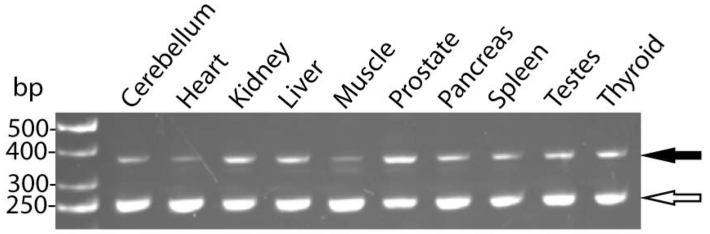

PCM1

Id	Gene Symbol	Inclusion Size	Exclusion Size	Exon Length
11	SMURF1	332	254	78
	RSPR	P_RSPR	Location in hg18	NT-AS
	4.8473	1E-05	chr7:98486474-98486551	
Left Primer		aacaacagtccagggcca		
Right Primer		ggctgggctccttgagtt		

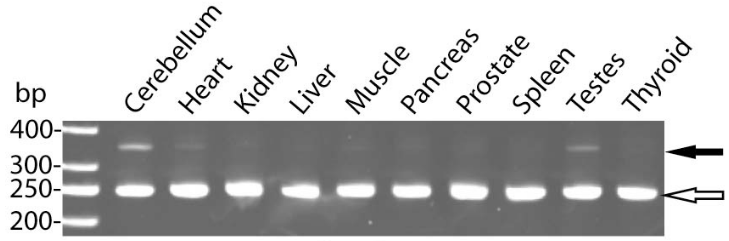

SMURF1

Id	Gene Symbol	Inclusion Size	Exclusion Size	Exon Length
12	LPHN1(Exon A)	265	250	15
	RSPR	P_RSPR	Location in hg18	T-AS
	49.9307	5E-05	chr19:14138828-14138842	
Left Primer		gcctttcctgacccctgt		
Right Primer		ccacgtagtcctcccacg		

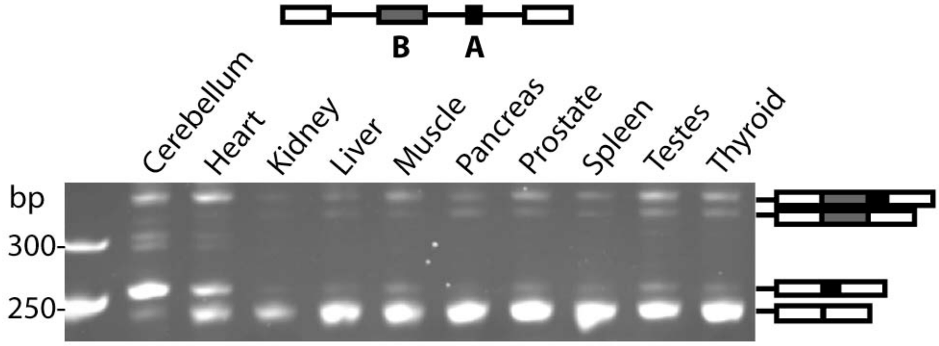

LPHN1

Id	Gene Symbol	Inclusion Size	Exclusion Size	Exon Length
13	DMWD	244	169	75
	RSPR	$P_{\_RSPR}$	Location in hg18	T-AS
	40.9557	9E-16	chr19:50979739-50979813	
Left Primer		catcatcactgcctgcca		
Right Primer		gcagggagggttatggct		

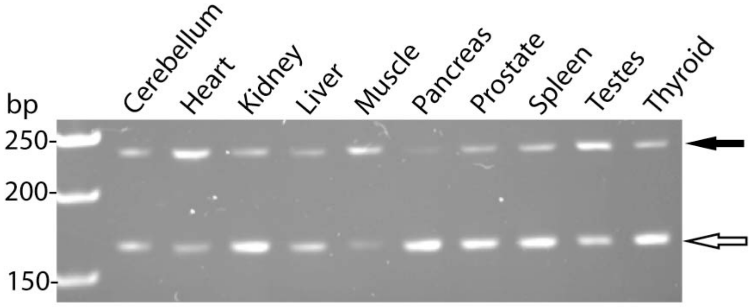

DMWD

Id	Gene Symbol	Inclusion Size	Exclusion Size	Exon Length
14	TCERG1	287	224	63
	RSPR	$P_{\_RSPR}$	Location in hg18	T-AS
	30.8229	1E-17	chr5:145828097-145828159	
Left Primer		acagccacacctgtgcaa		
Right Primer		gcaatagcaacctgggga		

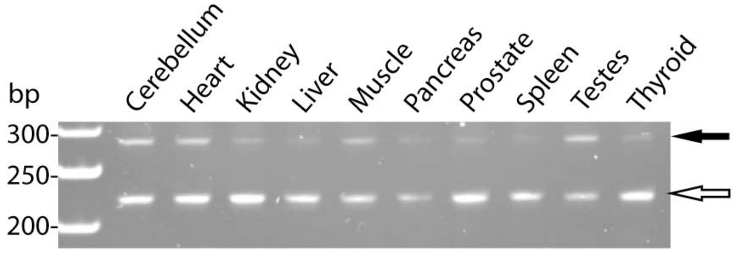

TCERG1

Id	Gene Symbol	Inclusion Size	Exclusion Size	Exon Length
15	RBM10	291	217	74
	RSPR	$P_{\_RSPR}$	Location in hg18	NT-AS
	27.5284	9E-16	chrX:46919362-46919435	
Left Primer		caggcagccactgaggat		
Right Primer		gaagcacttctctcggcg		

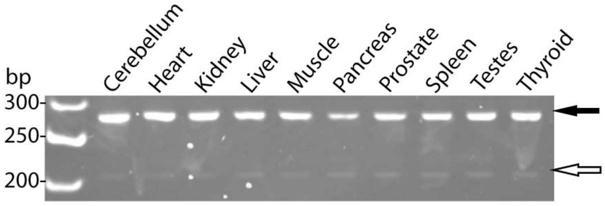

RBM10

Id	Gene Symbol	Inclusion Size	Exclusion Size	Exon Length
16	MAP2K7	245	197	48
	RSPR	$P_{\_RSPR}$	Location in hg18	T-AS
	24.9002	2E-14	chr19:7876693-7876740	
Left Primer		aagcaggagaaccgggag		
Right Primer		gtgaacagggttgacggg		

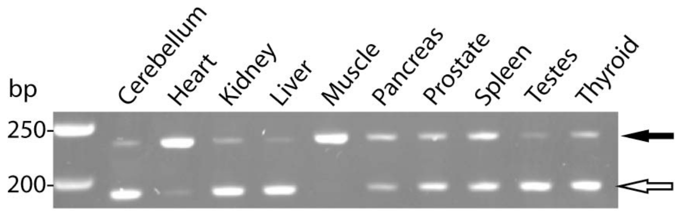

MAP2K7

Id	Gene Symbol	Inclusion Size	Exclusion Size	Exon Length
17	C1orf9	172	151	21
	RSPR	$P_{\_RSPR}$	Location in hg18	T-AS
	23.8038	6E-10	chr1:170813302-170813322	
Left Primer		ttcatggtagagatgagcgg		
Right Primer		tccaccatgctagtgcca		

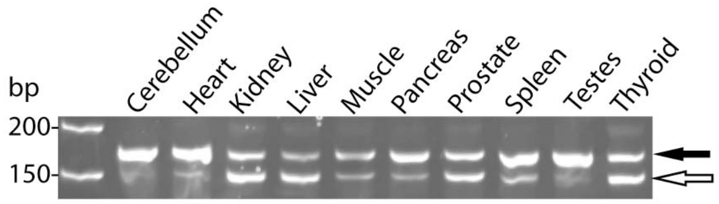

C1orf9

Id	Gene Symbol	Inclusion Size	Exclusion Size	Exon Length
18	LPHN1 (Exon B)	350	265	85
	RSPR	$P_{\_RSPR}$	Location in hg18	T-AS
	22.8582	4.76E-16	chr19:14136433-14136517	
Left Primer		gcctttcctgacccctgt		
Right Primer		ccacgtagtcctcccacg		

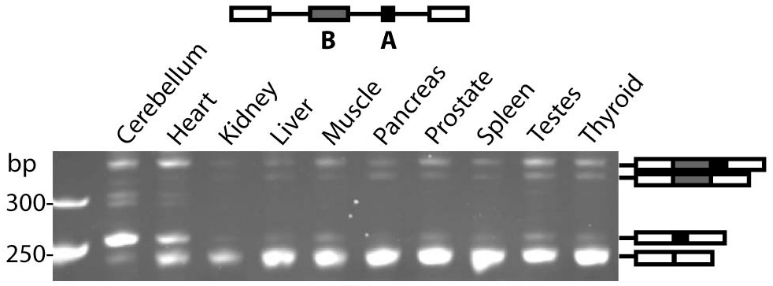

LPHN1

Id	Gene Symbol	Inclusion Size	Exclusion Size	Exon Length
19	IQSEC2	371	275	96
	RSPR	P_{RSPR}	Location in hg18	NT-AS
	21.0059	7E-10	chrX:53325471-53325566	
Left Primer		gtggagacaccggctagg		
Right Primer		cctcctcctcctgcttcc		

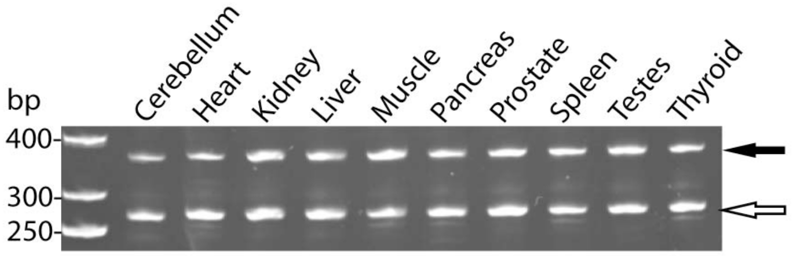

IQSEC2

Id	Gene Symbol	Inclusion Size	Exclusion Size	Exon Length
20	KIAA0433	509	335	174
	RSPR	P_{RSPR}	Location in hg18	T-AS
	20.0991	1E-41	chr5:102546834-102547007	
Left Primer		gggagccaaaggttgtga		
Right Primer		taaacaggcccgcacagt		

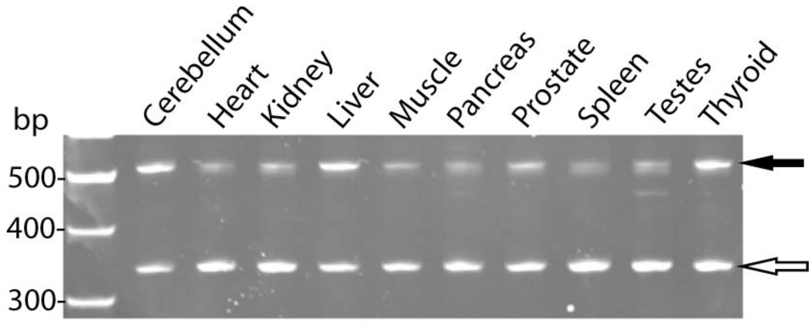

KIAA0433

Id	Gene Symbol	Inclusion Size	Exclusion Size	Exon Length
21	WSB2	340	239	101
	RSPR	$P_{\_RSPR}$	Location in hg18	Const
	0.0412	5E-04	chr12:116960476-116960576	
Left Primer		caggctcctgcttttga		
Right Primer		acaagcagggcagagtcg		

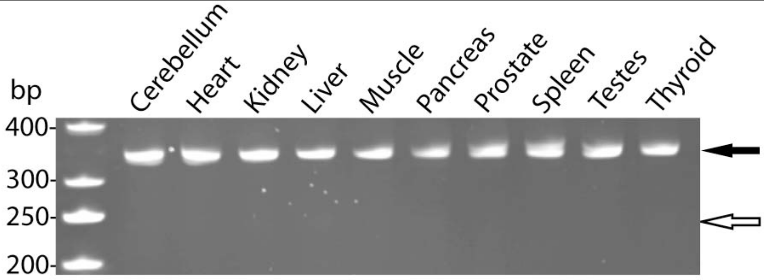

WSB2

Id	Gene Symbol	Inclusion Size	Exclusion Size	Exon Length
22	NGLY1	342	164	178
	RSPR	$P_{\_RSPR}$	Location in hg18	NT-AS
	0.5849	7E-05	chr3:25736509-25736686	
Left Primer		gatgggagaatggcgtgt		
Right Primer		tgtgttgccaagcgacat		

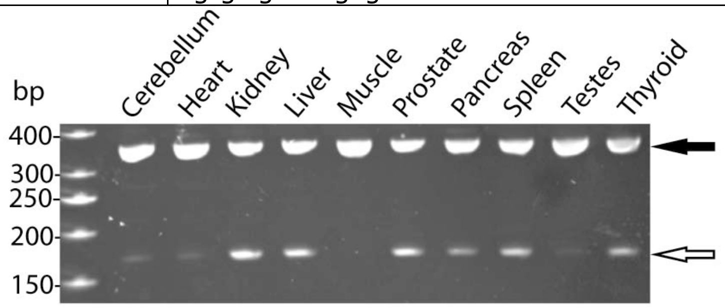

NGLY1

Id	Gene Symbol	Inclusion Size	Exclusion Size	Exon Length
23	DKFZp761L141	304	144	160
	RSPR	$P_{\_RSPR}$	Location in hg18	Const
	0.6355	5E-04	chr7:98295738-98295897	
Left Primer		cgctgactgctggatgtg		
Right Primer		cgccaccactttgagctt		

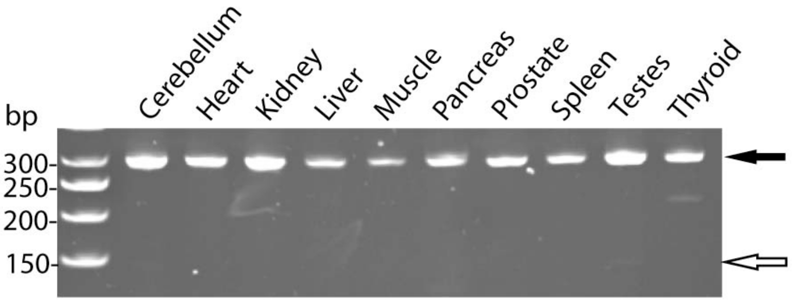

DKFZp761L141

Id	Gene Symbol	Inclusion Size	Exclusion Size	Exon Length
24	SLC17A3	319	231	88
	RSPR	$P_{\_RSPR}$	Location in hg18	T-AS
	0.7403	4E-05	chr6:25969831-25969918	
Left Primer		gcgagtggttggcatttc		
Right Primer		aaggcagcagacacagcc		

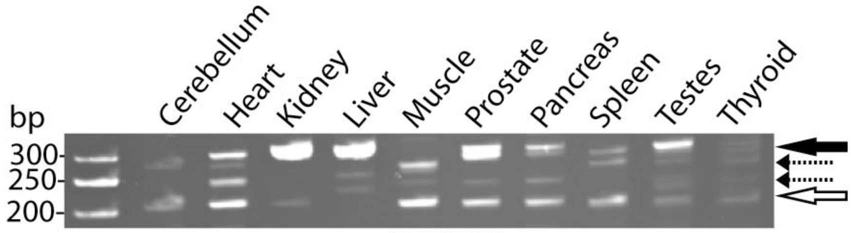

SLC17A3

Id	Gene Symbol	Inclusion Size	Exclusion Size	Exon Length
25	BRPF3	330	232	98
	RSPR	$P_{\_RSPR}$	Location in hg18	NT-AS
	0.7524	2E-05	chr6:36297923-36298020	
Left Primer		gagagcgaaggggagagg		
Right Primer		ggcagggtaggaggggta		

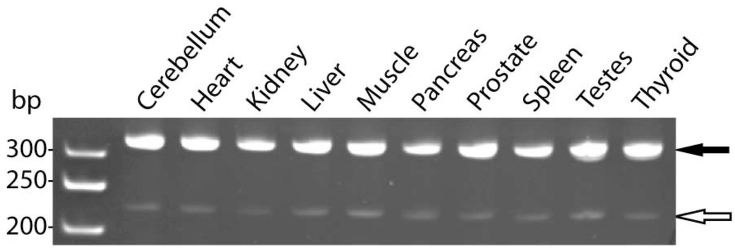

BRPF3

Id	Gene Symbol	Inclusion Size	Exclusion Size	Exon Length
26	GPNMB	409	235	174
	RSPR	$P_{\_RSPR}$	Location in hg18	NT-AS
	0.767	7E-04	chr7:23263036-23263209	
Left Primer		ccagtgactcaccagccc		
Right Primer		gcccgtccatgtcttctg		

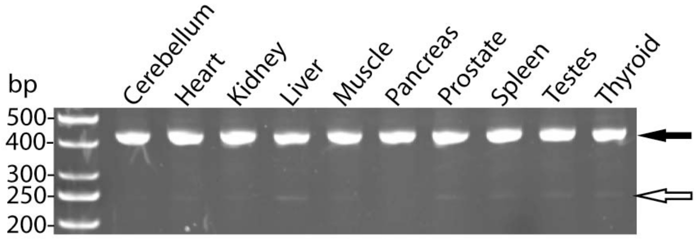

GPNMB

Id	Gene Symbol	Inclusion Size	Exclusion Size	Exon Length
27	DMTF1	388	159	229
	RSPR	P_RSPR	Location in hg18	Const
	0.7723	2E-06	chr7:86651649-86651877	
Left Primer		cggcgctaggaagaagtg		
Right Primer		agccattgtggtgaacgg		

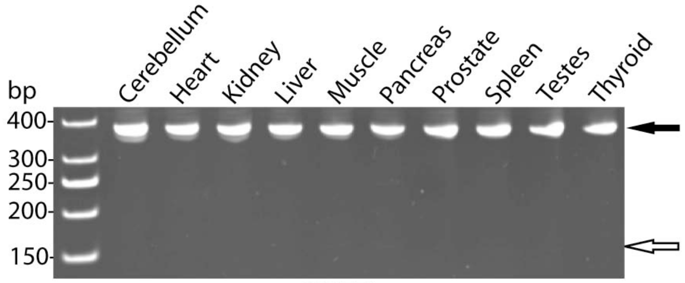

DMTF1

Id	Gene Symbol	Inclusion Size	Exclusion Size	Exon Length
28	MIR	567	386	181
	RSPR	P_RSPR	Location in hg18	Const
	0.7824	1E-04	chr10:45276685-45276865	
Left Primer		tccatggcccaaaatgac		
Right Primer		tggcaatgacgtggaatg		

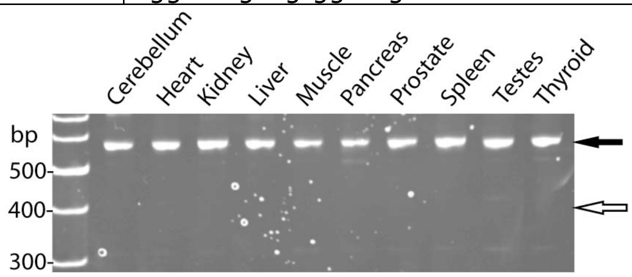

MIR

Id	Gene Symbol	Inclusion Size	Exclusion Size	Exon Length
29	HSGT1	519	301	218
	RSPR	P_RSPR	Location in hg18	NT-AS
	0.8006	8E-04	chr10:74593497-74593714	
Left Primer		atcagctctcgcagtcgg		
Right Primer		ttccccatggcagaaaaa		

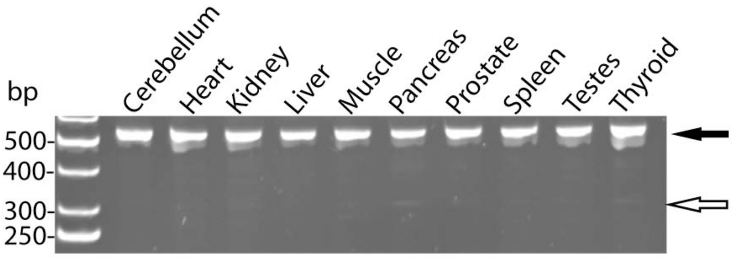

HSGT1

Id	Gene Symbol	Inclusion Size	Exclusion Size	Exon Length
30	POLDIP3	297	230	67
	RSPR	P_RSPR	Location in hg18	Const
	0.8072	2E-04	chr22:41313456-41313522	
Left Primer		ggtccatcctggggtagc		
Right Primer		acagaggcccctgaggac		

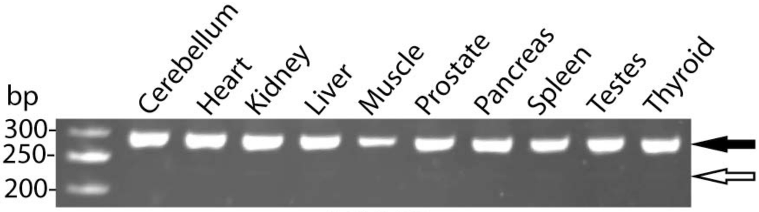

POLDIP3

Id	Gene Symbol	Inclusion Size	Exclusion Size	Exon Length
31	SORT1	397	273	124
	RSPR	P_RSPR	Location in hg18	Const
	0.5868	3E-04	chr1:109658406-109658529	
Left Primer		ccagggtggggtaaatcc		
Right Primer		tggagccacagggagtgt		

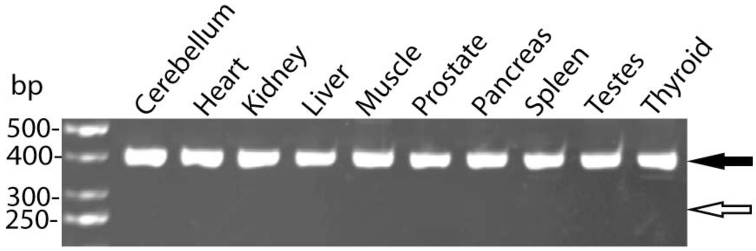

SORT1

Id	Gene Symbol	Inclusion Size	Exclusion Size	Exon Length
32	PHF7	304	182	122
	RSPR	P_RSPR	Location in hg18	NT-AS
	0.5934	8E-04	chr3:52431816-52431937	
Left Primer		acagagatgctgcctggg		
Right Primer		tcctcagggtggaaggtg		

PHF7-Hu(Inc 304 Skip 183) 64C (2009-08-17)

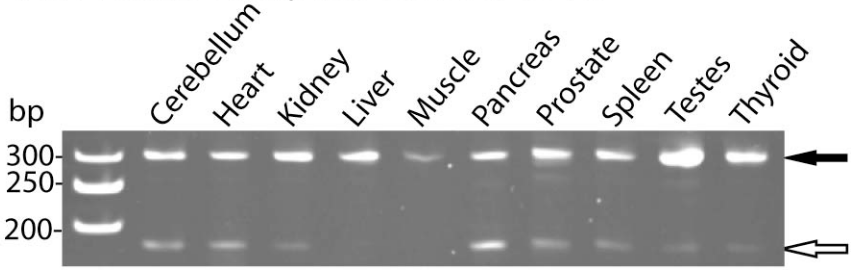

Id	Gene Symbol	Inclusion Size	Exclusion Size	Exon Length
33	RTKN	206	144	62
	RSPR	$P_{\_RSPR}$	Location in hg18	T-AS
	0.6035	3E-04	chr2:74511251-74511312	
Left Primer		agctacatgggcgagctg		
Right Primer		agcaggaacacagcccag		

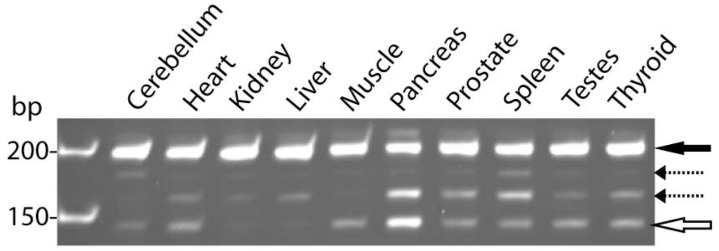

RTKN

Id	Gene Symbol	Inclusion Size	Exclusion Size	Exon Length
34	ATF6	308	231	77
	RSPR	$P_{\_RSPR}$	Location in hg18	NT-AS
	0.6121	4E-05	chr1:160014658-160014734	
Left Primer		tcacggagttccagggag		
Right Primer		tggctgaggttctgcctt		

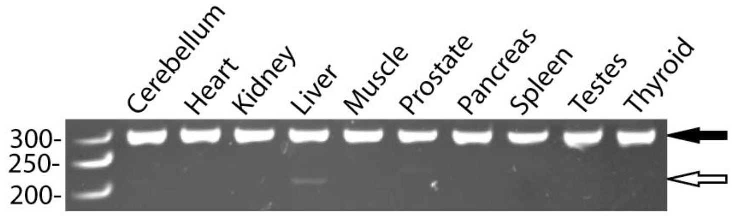

ATF6

Id	Gene Symbol	Inclusion Size	Exclusion Size	Exon Length
35	SCMH1	426/361	283/218	143
	RSPR	$P_{\_RSPR}$	Location in hg18	Const
	0.4599	6E-04	chr1:41266843-41266985	
Left Primer		cccaccaaccttgtcagc		
Right Primer		ctgcttcagccggtcaat		

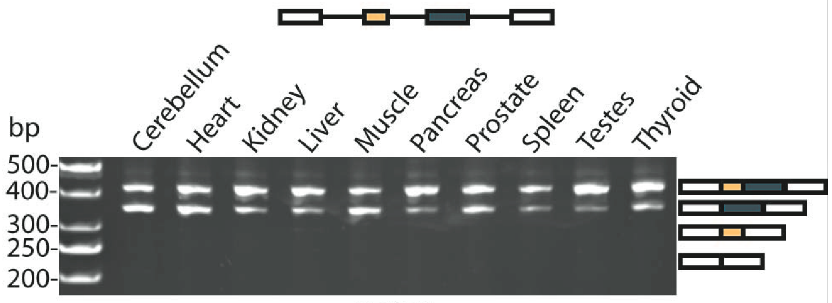

SCMH1

The target exon with RSPR annotated is in black.

Id	Gene Symbol	Inclusion Size	Exclusion Size	Exon Length
36	NECAP1	225	143	82
	RSPR	$P_{\_RSPR}$	Location in hg18	NT-AS
	0.4986	2E-04	chr12:8135632-8135713	
Left Primer		ttgctgtggagacggtga		
Right Primer		ccttgaagcccagatcca		

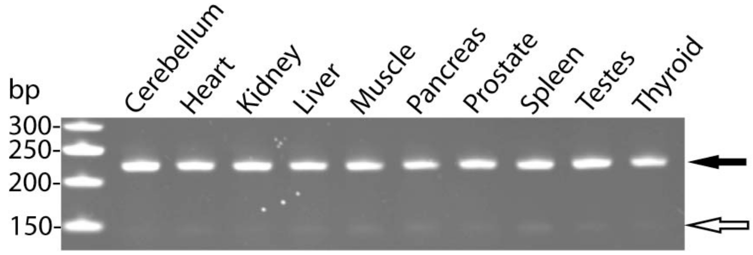

NECAP1

Id	Gene Symbol	Inclusion Size	Exclusion Size	Exon Length
37	SENP2	369	237	132
	RSPR	$P_{\_RSPR}$	Location in hg18	NT-AS
	0.5192	3E-06	chr3:186817962-186818093	
Left Primer		ctgggcagtggaagcaat		
Right Primer		tgcttggtaacccccaga		

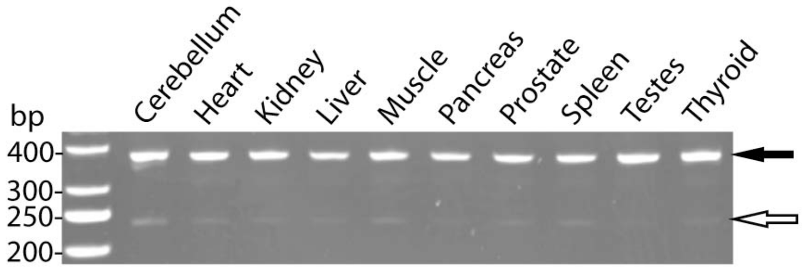

SENP2

Id	Gene Symbol	Inclusion Size	Exclusion Size	Exon Length
38	IFNAR1	328	186	142
	RSPR	$P_{\_RSPR}$	Location in hg18	Const
	0.5412	3E-05	chr21:33637711-33637852	
Left Primer		tgtgggctttggatggtt		
Right Primer		gcgtggagccactgaact		

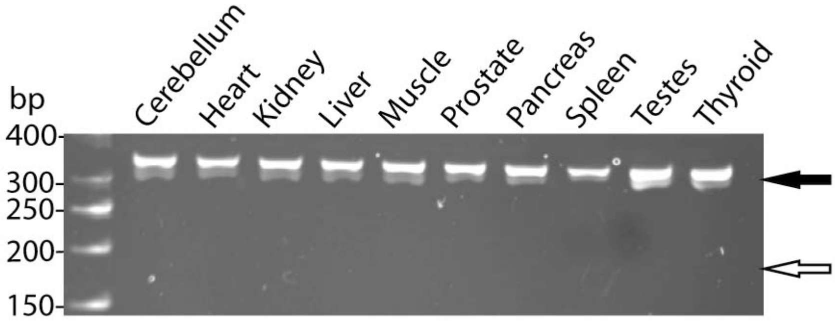

IFNAR

Id	Gene Symbol	Inclusion Size	Exclusion Size	Exon Length
39	PROM2	540	393	147
	RSPR	$P_{\_RSPR}$	Location in hg18	T-AS
	0.568	3E-06	chr2:95314444-95314590	
Left Primer		gtttgcagacaccccagg		
Right Primer		agattcggggcagaggac		

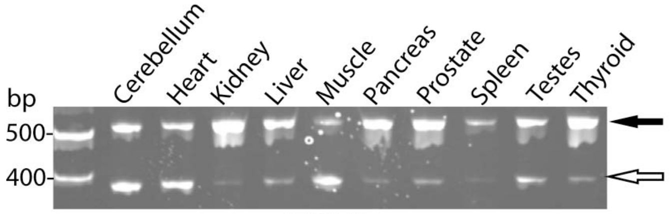

PROM2

Id	Gene Symbol	Inclusion Size	Exclusion Size	Exon Length
40	TARBP2	499	371	128
	RSPR	$P_{\_RSPR}$	Location in hg18	T-AS
	0.5744	9E-05	chr12:52185186-52185313	
Left Primer		agtctgagtgcaaccccg		
Right Primer		ccactcaggctcagctcc		

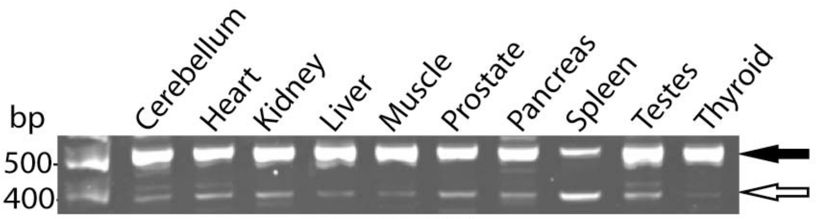

TARBP2
