# Supplementary material for: Predicting Functional Alternative Splicing by Measuring RNA Selection Pressure from Multigenome Alignments
Source: PLoS Comput Biol. 2009 Dec 18;5(12):e1000608. doi: 10.1371/journal.pcbi.1000608 (PMC2784930; doi:10.1371/journal.pcbi.1000608)

Table S5. RT-PCR Validation of 10 Brain-Specific Exons

We performed RT-PCR experiments to test a subset of the RSPR predictions. Tissue-specific splicing is one type of functional regulation that can be assayed easily in a panel of different tissue samples. We therefore extracted a subset of high RSPR exons that were also predicted by bioinformatics to up-regulated in brain based on available EST counts. We assayed their splicing by RT-PCR in cerebellum, heart, kidney and seven other human tissues. 80% (8/10) were found to be regulated in a tissue-specific manner. Thus a large fraction of high-RSPR exons were validated by experimental evidence of functional regulation.

A, Exons

GENE	EXON LOCATION IN hg18	RSPR	$P_{\_RSPR}$	RT-PCR RESULT
EXOC7	chr17:71594530-71594568	6.24	$1.6X10^{-10}$	Brain specific exon inclusion
PLEKHB1	chr11:73044496-73044600	4.01	$3.6X10^{-08}$	No change in splicing observed
FLJ11730	chr1:37734895-37734924	6.07	$4.0X10^{-08}$	Brain specific exon inclusion
LYK5	chr17:59154390-59154418	25.37	$1.1X10^{-11}$	Brain specific exon inclusion,heart and muscle to a lesser degree
TBC1D1	chr4:37731122-37731241	6.42	$4.2X10^{-28}$	Brain, heart, muscle and testes specific exon inclusion
SEC3L1	chr4:56427795-56427815	6.60	$1.8X10^{-06}$	Brain specific exon inclusion
FLJ13611	chr5:64987219-64987236	47.94	$3.3X10^{-09}$	Brain specific exon skipping
DCTN4	chr5:150091641-150091661	8.62	$3.1X10^{-10}$	Brain specific exon inclusion
G3BP2	chr4:76798191-76798289	6.68	$3.6X10^{-18}$	Brain specific exon inclusion,heart and muscle to a lesser degree
AP2A1	chr19:54997603-54997668	5.15	$1.0X10^{-08}$	Brain specific exon inclusion,heart and muscle to a lesser degree

B, Primers

GENE NAME	LEFT PRIMER	RIGHT PRIMER
EXOC7	GACCCGGACAAGGAGTACAA	TCTCCAGGGACTTGAGGATG
PLEKHB1	ACTGCTGGAGGCAAACTCC	AGTAGCTGCGGACATACGTG
FLJ11730	GATGGATCTGTGCAGGGAGT	GAGGTCAGAGAAGGGAAGCA
LYK5	GTACTCCCCTCCCAGCAAC	CTCGTAACACCCTCCCTCTG
TBC1D1	GCCATCTGTGTGTGAAAAGG	AGGAATATCTGCTGCCTCCA
SEC3L1	TATAAATGGGTTGCCAGCAG	TGCAGCTCTCTGGACAATTT
FLJ13611	AACCATTGGATGTGAAAACC	TCCAGCTTGGCTGACTGAAT
DCTN4	AGATCGCAAGAAACTGGCAC	GGCAAGGGTACTGATGGATG
G3BP2	CTACTACTCCTCCTCCGGCA	CCAGGTCGTTCTCTAGGTCG
AP2A1	CAGGGAACCTTCTGGTGGA	ATTCAGCAACTCATCGGCTT

C, Result,

The target exon with RSPR annotated is in black.

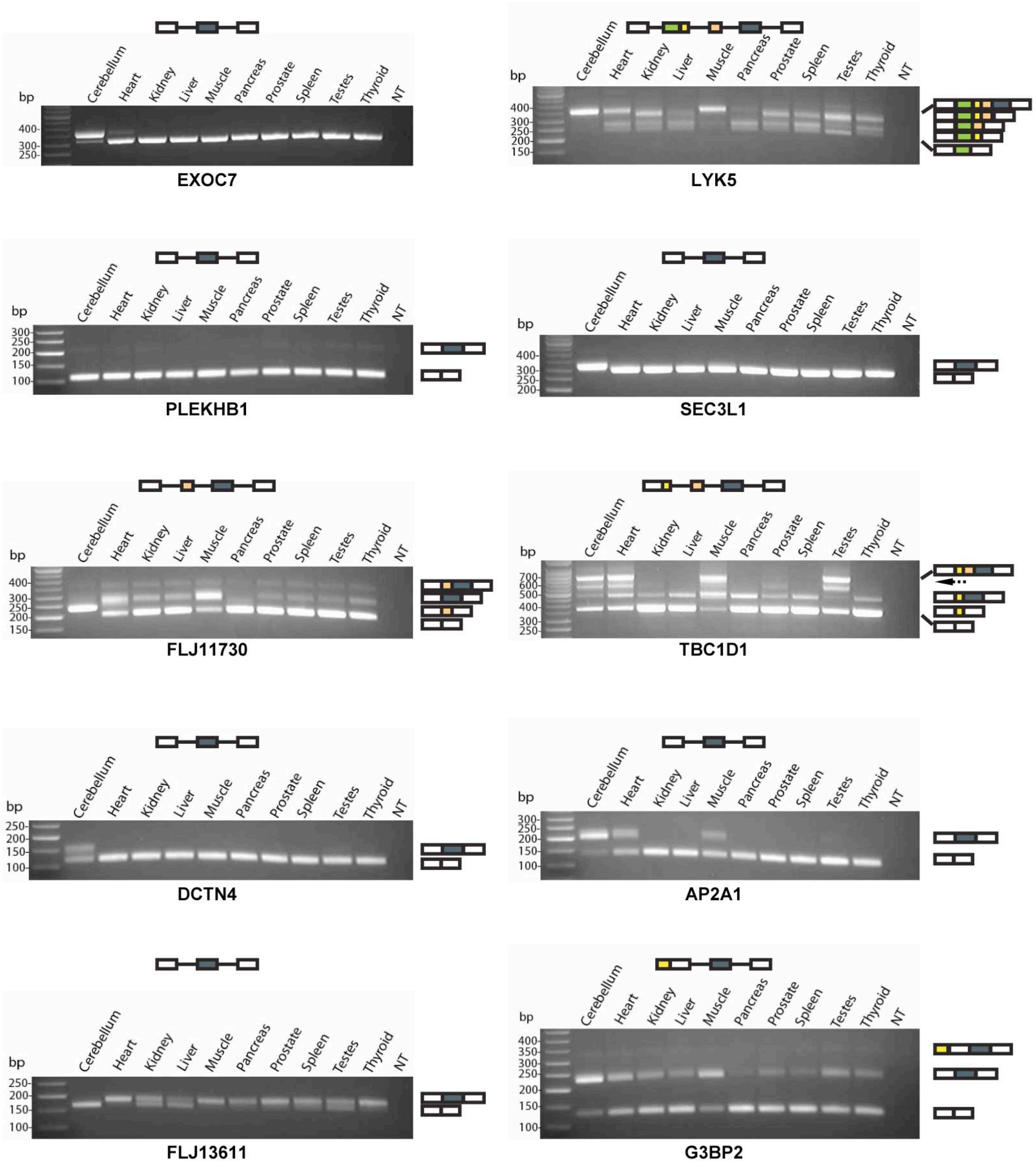

Supplement: Table S5 — RT-PCR Validation of 10 Brain-Specific Exons. We performed RT-PCR experiments to test a subset of the RSPR predictions. Tissue-specific splicing is one type of functional regulation that can be assayed easily in a panel of different tissue samples. We therefore extracted a subset of high RSPR exons that were also predicted by bioinformatics to up-regulated in brain based on available EST counts. We assayed their splicing by RT-PCR in cerebellum, heart, kidney and seven other human tissues. 80% (8/10) were found to be regulated in a tissue-specific manner. Thus a large fraction of high-RSPR exons were validated by experimental evidence of functional regulation. (1.09 MB PDF) [file pcbi.1000608.s005.pdf]
