# Supplementary material for: Predicting Functional Alternative Splicing by Measuring RNA Selection Pressure from Multigenome Alignments
Source: PLoS Comput Biol. 2009 Dec 18;5(12):e1000608. doi: 10.1371/journal.pcbi.1000608 (PMC2784930; doi:10.1371/journal.pcbi.1000608)
Supplement: Table S6 — Alternative Exons in Exoc7. RSPR results for AS exons in Exoc7. TISSUE LOD: Log Odds score (log-base 10) for tissue-specific AS in the specified tissues. (0.07 MB PDF) [file pcbi.1000608.s006.pdf]

Table S6, Alternative exons in Exoc7.

EXON_ID*	RSPR	$P_{\_RSPR}$	TISSUE LOD*	LOCATION IN STRUCTURE
25161	6.24	1.67×10^{-10}	brain_nerve : 2.5<br>retina : 2.2	Between H12-H13
25162	1.02	1.30×10^{-2}	-	
25152	5.35	3.94×10^{-20}	breast : 3.95	Between H6-H7
25153	1.77	7.68×10^{-6}	-	
25154	2.15	2.50×10^{-12}	-	

**EXON_ID and TISSUE LOD are defined in ASAPII.*
