# Supplementary figures and images for: Predicting Functional Alternative Splicing by Measuring RNA Selection Pressure from Multigenome Alignments
Source: PLoS Comput Biol. 2009 Dec 18;5(12):e1000608. doi: 10.1371/journal.pcbi.1000608 (PMC2784930; doi:10.1371/journal.pcbi.1000608)

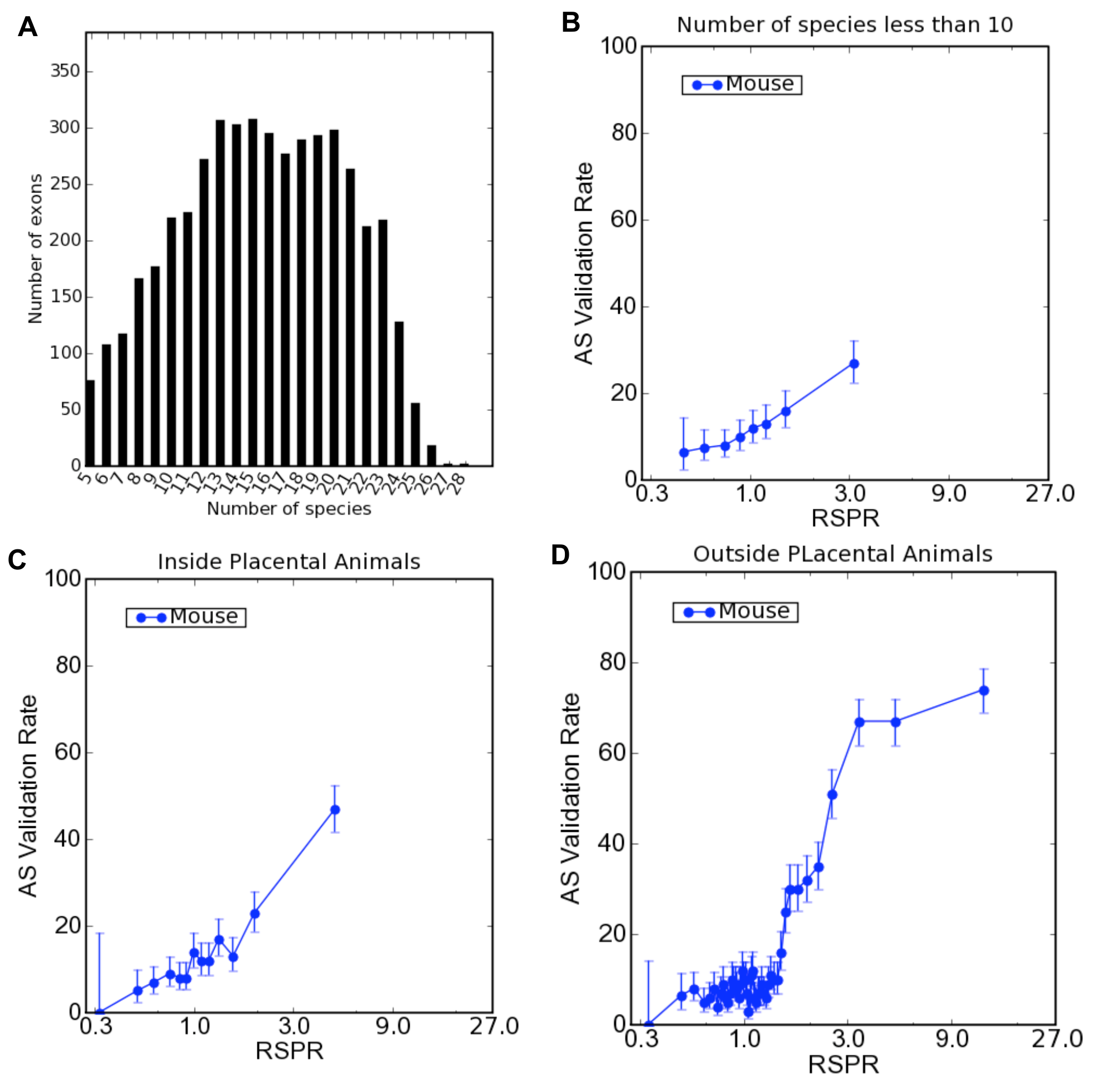

Supplement: Figure S1 — Analysis of the species used for RSPR calculations on human AS exons. (A) A histogram of the number of species used for each RSPR calculation. (B–D) The fraction of exons that were observed to be alternatively spliced in independent mouse EST data (y-axis), as a function of RSPR (x-axis), for (B) the set of RSPR calculations with 5–9 species; (C) the set of RSPR calculation that used only placental mammal species; (D) the set of RSPR calculations that used both placental mammals and other vertebrates. (0.61 MB TIF) [file pcbi.1000608.s007.tif]

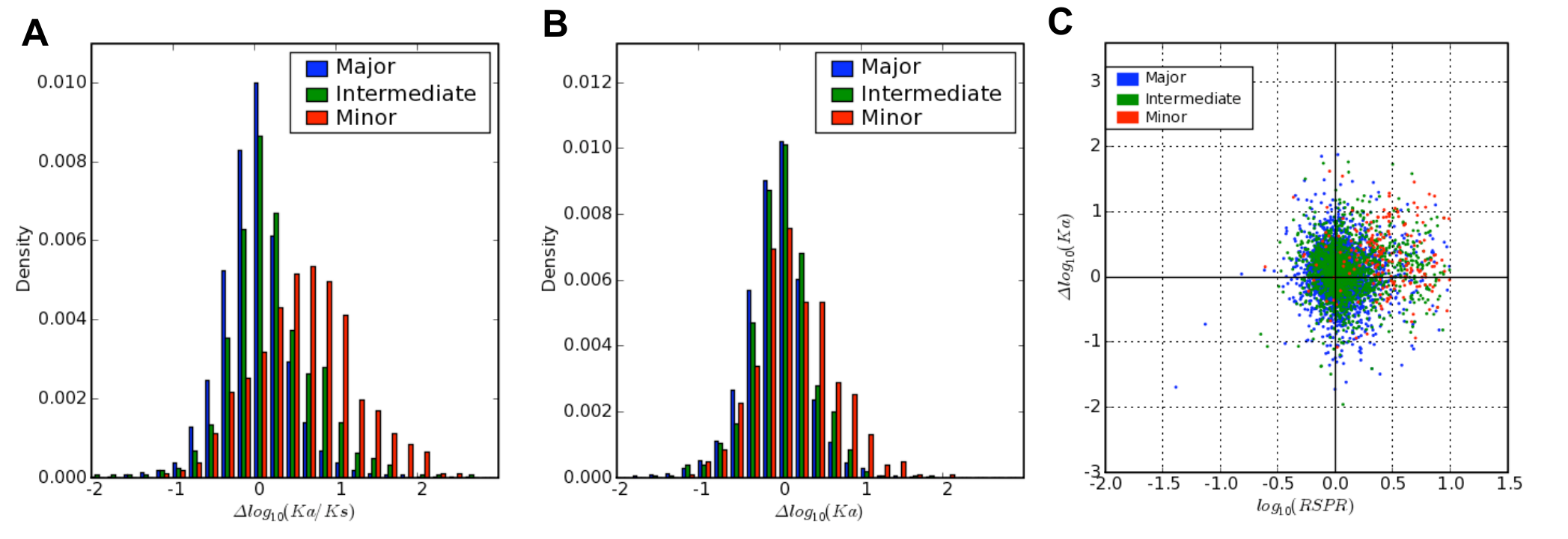

Supplement: Figure S2 — Ka/Ks and Ka distributions for different exon inclusion levels. For each alternative exon, we calculated the log-difference for its Ka/Ks or Ka relative to its control region (constitutive exons in the same gene). We then plotted their distributions for minor form, major form, and intermediate form alternative exons. (A) Histograms of the amino acid selection pressure (Ka/Ks); (B) Histograms of the non-synonymous mutation rate (Ka); (C) A scatter plot of the non-synonymous mutation rate (y-axis) vs. RSPR (x-axis). (0.43 MB TIF) [file pcbi.1000608.s008.tif]

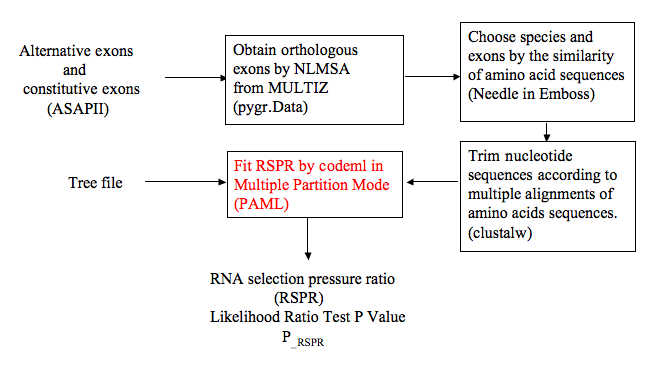

Supplement: Figure S3 — A flow chart of the RNA selection pressure ratio (RSPR) calculation. (0.07 MB TIF) [file pcbi.1000608.s009.tif]
